# Dectin-1 is Pathogenic in Chronic Kidney Disease by Promoting Macrophage Infiltration and Transition to Myofibroblast

**DOI:** 10.7150/ijbs.119129

**Published:** 2025-08-16

**Authors:** Lingling Shen, Jingyi Li, Anqi Zhang, Sijing Yan, Wenxin Sha, Yucheng Wang, Shi Feng, Cuili Wang, Zhimin Chen, Hongfeng Huang, Bingjue Li, Pingping Ren, Suhan Zhou, Siqi Wu, Yanli Wang, Zhouji Shen, Song Rong, Hermann Haller, Hong Jiang, Jianghua Chen

**Affiliations:** 1 Kidney Disease Center, the First Affiliated Hospital, College of Medicine, Zhejiang University, Hangzhou, Zhejiang, 310000, China.; 2 Institute of Nephropathy, Zhejiang University, Hangzhou, Zhejiang, 310000, China.; 3 Zhejiang Clinical Research Center of Kidney and Urinary System Disease, Hangzhou, Zhejiang, 310000, China.; 4 The First Clinical School of Medicine, Zhengzhou University, Zhengzhou, Henan, China.; 5 Department of Pathology, the First Affiliated Hospital, College of Medicine, Zhejiang University, Hangzhou, Zhejiang, 310000, China.; 6 Ningbo Medical Center LiHuiLi Hospital, The Affiliated LiHuiLi Hospital of Ningbo University, China.; 7 Department of Nephrology and Hypertension, Hannover Medical School, Hannover, Germany.

## Abstract

Dectin-1, a pattern recognition receptor predominantly expressed on myeloid cells, is required for maintaining immune homeostasis. However, the role of Dectin-1 in chronic kidney disease (CKD) remains unknown. Here we reported that Dectin-1 was markedly upregulated in the fibrotic kidneys of CKD patients, primarily in macrophages, and its expression correlated with fibrosis severity and renal dysfunction. Genetic deletion of Dectin-1 attenuated renal fibrosis induced by unilateral ureteral obstruction (UUO) or ischemia-reperfusion (IR), a finding confirmed in bone marrow chimeric mice. Macrophage-specific Dectin-1 deletion similarly protected against renal fibrosis, demonstrating its cell-autonomous role. Mechanistically, Dectin-1 promoted macrophage infiltration via Syk/NF-κB/CCL2-CCR2 axis, while facilitating macrophage-to-myofibroblast transition (MMT) by activating TGF-β/Smad signaling. Pre-clinically, pharmacological inhibition of Dectin-1 with Laminarin significantly reduced renal fibrosis in UUO and IR models, highlighting its therapeutic potential for CKD.

## Introduction

Chronic kidney disease (CKD) poses a considerable and growing global health challenge, affecting roughly 10% of the adult population worldwide 1, 2. Regardless of the initial cause, nearly all types of CKD will ultimately result in renal fibrosis, which is characterized by the infiltration of inflammatory cells, activation and proliferation of fibroblasts, extensive production and deposition of extracellular matrix (ECM) components, as well as tubular atrophy and microvascular rarefaction 3, 4. However, current therapies offer limited effectiveness, only slowing disease progression 5. Therefore, a deeper understanding of the molecular mechanisms underlying renal fibrosis is essential for developing novel therapeutic strategies.

Macrophages are known to play a crucial role in regulating renal inflammation and fibrosis through the production of pro-inflammatory cytokines and pro-fibrotic factors 6, 7. Previous research has shown that the macrophages accumulated in fibrotic kidneys are primarily recruited from the bone marrow (BM), while resident macrophages contribute minimally to renal fibrosis 8. Furthermore, BM-derived macrophages serve as a key source of renal myofibroblasts, which are characterized by the expression of α-smooth muscle actin (α-SMA) and are the predominant cell type responsible for ECM production during renal fibrosis 9, 10.

Dectin-1, encoded by *CLEC7A*, is a member of the C-type lectin family of pattern recognition receptors and is mainly expressed on myeloid-monocytic lineage cells, such as macrophages 11. Originally, Dectin-1 was discovered to recognize β-glucans in fungal pathogens, and then recruits and phosphorylates spleen tyrosine kinase (Syk), which subsequently activates nuclear factor-κB (NF-κB), thereby initiating an anti-fungal immune response 12, 13. Recent studies have revealed that Dectin-1 is also implicated in non-infectious diseases, including ischemia-reperfusion injury 14, autoimmune disorders 15, 16, allergies 17, 18, and cancer 19, 20. In non-infectious kidney diseases, Dectin-1 was shown to mediate Ang II-induced renal injury through the activation of neutrophil migration and TGF-β1 secretion 21. However, whether Dectin-1 contributes to unilateral ureteral obstruction (UUO) or ischemia-reperfusion (IR)-induced renal fibrosis is largely unknown.

In this study, we examined the expression of Dectin-1 in the fibrotic kidneys of CKD patients and mouse models, and characterized its functional involvement in renal fibrosis induced by UUO or IR. Our findings demonstrate that Dectin-1 is primarily expressed on macrophages and plays a pathogenic role in renal fibrosis by promoting macrophage infiltration and macrophage-to-myofibroblast transition (MMT).

## Materials and Methods

### Human renal biopsy samples

Renal biopsy samples were obtained from 120 CKD patients, including 84 IgA nephropathy, 6 diabetic kidney disease, 6 hypertensive nephropathy, 6 lupus nephritis, 6 minimal change disease, 6 membranous nephropathy, and 6 mesangial proliferative glomerulonephritis, over periods of June 2021 to June 2022 from the Department of Pathology, Kidney Disease Center at the First Affiliated Hospital, Zhejiang University School of Medicine (Supplementary Table S1). All patients provided the written informed consent before participation. This study was approved by the Research Ethics Committee of the First Affiliated Hospital, Zhejiang University School of Medicine (No. 2023-109).

### Mouse models

*Clec7a* knockout (Dectin-1 KO) mice on a C57BL/6 background were generously provided by Professor Guang Liang from Hangzhou Medical College and were determined by genotyping as described previously 22. Mice with macrophage-specific *Clec7a* deletion in C57BL/6 mice was achieved using the Cre-loxP system. Dectin-1-flox and Cd68-2A-Cre mice, obtained from Shanghai Model Organisms, were bred to generate Cre-positive mice for macrophage-specific *Clec7a* KO (Dectin-1^△Cd68^) and Cre-negative littermate controls (Dectin-1^f/f^) determined by genotyping with primers as follows: Dectin-1-flox Forward Primer: TGCTGTGTAATTTTCAAAGAGCTA; Dectin-1-flox Reverse Primer: AGGGGATGCCTATGGTTATTGA; Cd68-2A-Cre Mutant Primer: GCAGAAGGGGCAGCCACACCAT; Cd68-2A-Cre Wild Type Primer: CTTTTAGCCCCGGCAGGACAA; Cd68-2A-Cre Common Primer: GCATCACCCGCAGACGACAATC. Male mice aged at 8-10 weeks were used for all experiments. All mice were housed at the Zhejiang University Animal Center following institutional animal care regulations. The mice experimental protocols were approved by the Research Ethics Committee of the First Affiliated Hospital, Zhejiang University School of Medicine (No.2023-136).

The UUO is a well-recognized CKD mouse model and was used in this study. As previously described 23, mice were anesthetized by inhalation of 2% isoflurane via inhalation. The left ureter was ligated twice using a 5/0 silk thread to induce obstruction. The sham control mice followed the surgery procedure without ligation of the left ureter. The left kidney was harvested at different time points post-surgery according to the experimental requirements.

For the folic acid (FA)-induced renal fibrosis model, mice were administered with a single intraperitoneal injection of folic acid (250 mg/kg, dissolved in 300 mM NaHCO_3_). Control mice received an equivalent volume of 300 mM NaHCO_3_. Kidney samples were collected at 14 days post-administration for various analyses.

An ischemia-reperfusion (IR) mouse model with chronic renal fibrosis was also used in this study. Briefly, mice were anesthetized with 2% isoflurane via inhalation. The left renal pedicles were exposed and clamped for 30 minutes using nontraumatic microvascular clips. Afterward, the clips were removed to initiate reperfusion. The contralateral kidneys were left untreated. Throughout the procedure, the body temperature of the mice was maintained between 36°C and 37°C using an electric heating blanket. Sham-operated mice served as controls. The left kidney was harvested at 14 days for examination after surgery.

To determine the role of bone marrow-derived Dectin-1 expressing cells in renal fibrosis, a mouse chimeric model was used. Briefly, after being irradiated with 9 Gy, mice were intravenously transferred with 2×10⁷ bone marrow cells (BMC) obtained from WT or Dectin-1 KO mice. Four weeks after BMC transfer, chimeric mice were validated by flow cytometry of peripheral blood. The role of bone marrow-derived Dectin-1-expressing cells in renal fibrosis was then investigated using the UUO model.

### Reagents and Antibodies

The reagents used in this study, included the Dectin-1 antagonist laminarin (L9634, Sigma, USA), the Dectin-1 agonist depleted zymosan (d-zymosan, tlrl-zyd, InvivoGen, USA), the spleen tyrosine kinase (Syk) inhibitor R406 (S2194, Selleck, USA), the NF-κB inhibitor PDTC (P8765, Sigma, USA), the recombinant mouse transforming growth factor-β1 (TGF-β1, 7666-MB, R&D System, USA), mouse tumor necrosis factor-alpha (TNF-α) ELISA kits (430907, BioLegend, USA), mouse ELISA kits for C-C motif chemokine ligand 2 (CCL2, MJE00B, R&D Systems, USA), TGF-β1 (DB100C, R&D Systems, USA), interleukin (IL)-6 (M6000B, R&D Systems, USA), and IL-1β (MLB00C, R&D Systems, USA).

We also acquired the following antibodies from Cell Signaling Technology (Beverly, USA) including: Dectin-1 (23042), Vimentin (5741), collagen type I (COL1a1, 72026), α-smooth muscle actin (α-SMA, 19245), Smad2/3 (8685), phospho (p-)Smad2/3 (8828), Syk (13198), p-Syk (2715), NF-κB p65 (8242), p-NF-κB p65 (3033), F4/80 (70076), and Lamin A/C (4777). We purchased additional antibodies from Thermo Fisher Scientific (Waltham, USA): Dectin-1 (PE-Cyanine7, 25-5859-80), CD45 (PE-Cyanine7, 25-0451-82), CD-11b (APC, 17-0112-82), F4/80 (FITC, 11-4801-85), Ly-6G (FITC, 11-9668-82), CD-11c (PE, 12-0114-83), MHC II (APC, 17-5321-82), p-Syk (PE, 12-9014-42), iNOS (PE, 12-5920-80), and CD206 (PE, 12-2061-82).

From BioLegend (California, USA), we obtained antibodies against CD45.1 (PE-Cyanine7, 110730), CD45.2 (Brilliant Violet 510, 109838), Ki-67 (PE-Cyanine7, 652426), and C-C motif chemokine receptor 2 (CCR2, PE-Cyanine7, 150612). We acquired antibodies from HUABIO (Hangzhou, China) for CCL2 (EM1710-22), IκBα (ET1603-6), p-IκBα (ET1609-78), and TGF-β1 (HA721143). Additional antibodies from Abcam (Cambridge, USA) including Dectin-1 (ab140039), fibronectin (ab2413), α-Tubulin (ab176560), and CD68 (ab201340). Also, antibodies against α-SMA (PE, IC1420P) from R&D Systems (Minnesota, USA), antibodies against p-Smad2/3 (PE, 562586) from BD Biosciences (New Jersey, USA), and antibodies against GAPDH (60004-1-Ig) from Proteintech (Chicago, USA).

### Histology, Immunohistochemistry, and Immunofluorescence

Mice were euthanized and perfused with saline. Their kidneys were harvested, fixed in 4% paraformaldehyde, embedded in paraffin, cut into 4-micron sections, and stained with Hematoxylin & Eosin (H&E) and Masson's Trichrome (Solarbio, China) for routine histological analysis and evaluation of renal fibrosis and immunohistochemistry with various antibodies as described previously 23.

For immunofluorescence staining, 8-μm thick OCT-embedded sections were incubated overnight with primary antibodies, followed by Alexa Fluor 488- or 594-conjugated secondary antibodies (Abcam, USA). A mounting medium containing DAPI was used for cell nucleus staining and slide mounting. For each kidney sample, at least five random fields were examined.

### Real-time quantitative polymerase chain reaction (RT-qPCR)

Total RNA was extracted from kidney tissues using TRIzol (Invitrogen, CA, USA) and reverse transcribed into cDNA with PrimeScript II Reverse Transcriptase (Takara, Shiga, Japan). RT-qPCR was conducted using SYBR Green on a CFX96 Real-Time PCR Detection System (Bio-Rad, CA, USA). Primer sequences are listed in Supplementary Table S2. Each transcript's average threshold cycle (Ct) was measured in triplicate. Relative mRNA expression was normalized to *Gapdh* and analyzed using the 2^-△△CT^ method.

### Flow cytometry

To generate single-cell suspensions, kidneys were harvested, finely minced, and enzymatically digested in PBS containing 1 mg/mL Collagenase A (Roche, Switzerland) and 100 U/mL DNase I (Roche, Switzerland) for 30 minutes at 37 °C. The digestion mixture was filtered through a 70 μm cell strainer (Falcon, USA). After erythrocyte lysis, cells were counted and resuspended in PBS at a final density of 1×10⁶ cells per 100 μL.

Single-cell suspensions were first incubated with an Fc blocker (Thermo Fisher Scientific, USA) at 4 °C for 20 minutes. For surface antigen staining, cells were treated with an antibody mixture at 4 °C in the dark for 30 minutes. For intracellular staining, cells were fixed in Fixation Buffer (Thermo Fisher Scientific, USA) at room temperature in the dark for 20 minutes. To remove residual Fixation Buffer, cells were washed twice with Intracellular Staining Perm Wash Buffer (Thermo Fisher Scientific, USA) at room temperature for 5 minutes per wash. Intracellular antigens were stained using antibody cocktails in Intracellular Staining Perm Wash Buffer in the dark for 20 minutes. Flow cytometry was performed on a BD FACSCanto II flow cytometer (BD Biosciences, USA), and data were analyzed with FlowJo_V10.

### Western Blot

We lysed kidney tissues and cells in RIPA buffer containing protease and phosphatase inhibitors. Protein concentration was measured using the BCA Protein Assay Kit (Thermo Fisher Scientific, USA). Proteins were separated via SDS-PAGE and transferred onto PVDF membranes (Millipore, USA). The membranes were blocked with 5% milk and incubated overnight at 4 °C with primary antibodies, followed by incubation with horseradish peroxidase-conjugated secondary antibodies. We detected target proteins using chemiluminescence with the Bio-Rad ChemiDoc MP system.

### Cell culture

We isolated bone marrow-derived macrophages (BMDMs) from the femurs and tibias of WT and Dectin-1 KO mice. Cells were cultured and differentiated for five days in RPMI-1640 medium supplemented with 10% FBS, 1% penicillin-streptomycin (P/S), and 50 ng/mL macrophage-stimulating factor (M-CSF).

Renal macrophages were isolated from the fibrotic kidneys of UUO-induced WT and Dectin-1 KO mice using the Mouse F4/80 Positive Selection Kit (STEMCELL, Canada) according to the manufacturer's instructions. We confirmed the purity of the sorted macrophages via flow cytometry. The isolated macrophages were cultured in RPMI-1640 medium containing 10% FBS and 50 ng/mL M-CSF. After 24 hours of incubation, we harvested the cells and collected the culture medium for further analysis.

Human kidney 2 (HK-2) proximal tubular cells were obtained from ATCC and cultured in DMEM supplemented with 10% FBS and 1% P/S.

### Enzyme-linked immunosorbent assay (ELISA)

We quantified CCL2, TNF-α, IL-6, IL-1β, and TGF-β1 concentrations using commercial ELISA kits, following the manufacturer's instructions.

### Data analysis

Data were statistically analyzed using SPSS 23 and GraphPad Prism 8. Normally distributed data are presented as mean ± SD and were compared using the *t*-test. Non-normally distributed data are expressed as medians (interquartile ranges) and analyzed with non-parametric tests. We assessed correlations between numerical variables using Pearson's correlation analysis. A *P*-value < 0.05 was considered statistically significant.

## Results

### Dectin-1 is highly upregulated in fibrotic kidneys of CKD patients and positively correlated with renal fibrosis

**Figure 1:**
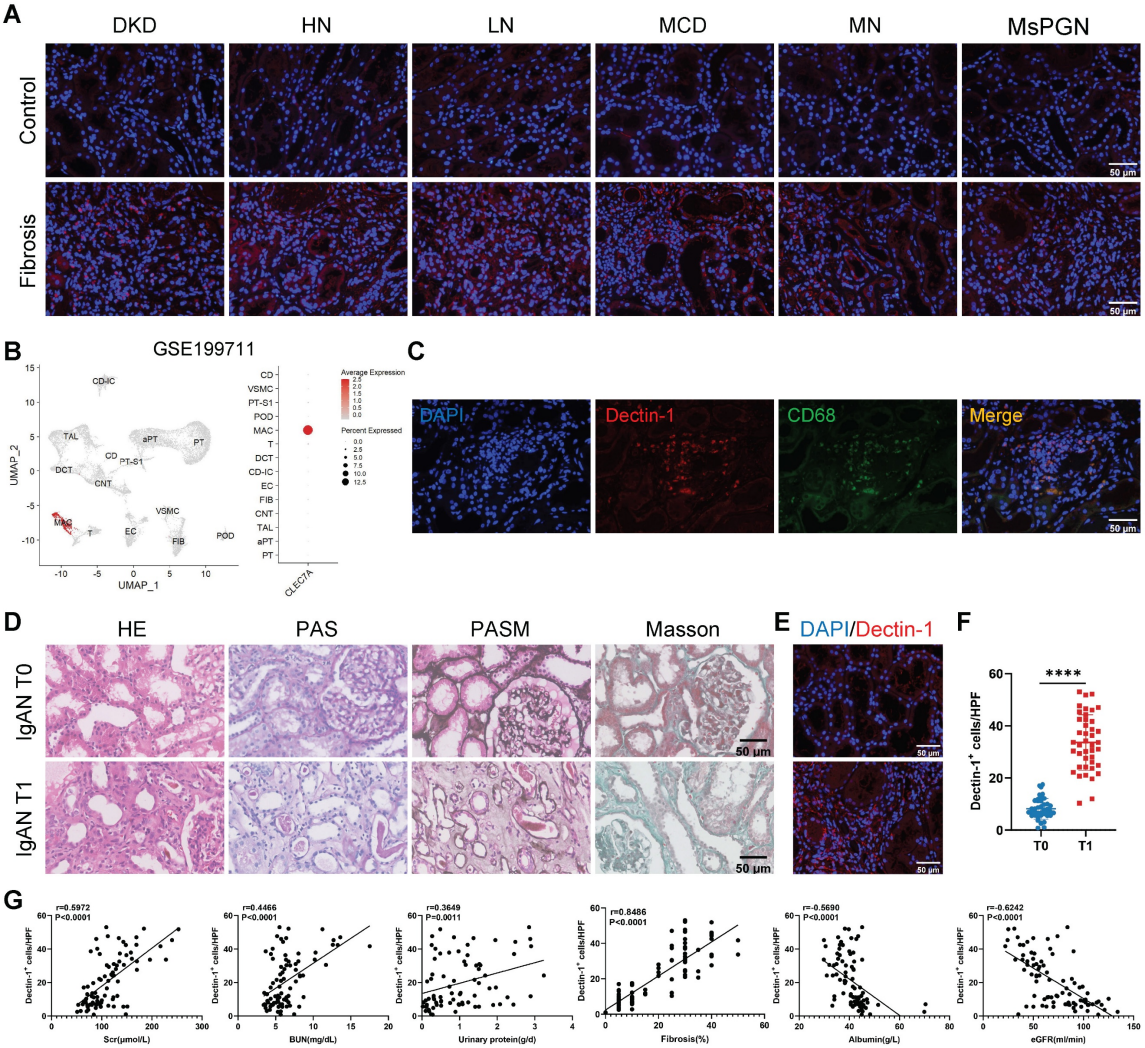
**Dectin-1 is markedly upregulated in the chronic kidney disease (CKD), presumably by macrophages, and is correlated significantly with declined renal function and progressive renal fibrosis.** (A) Representative photomicrographs of Dectin-1 (red) immunofluorescence staining in renal cortical tissue from patients with diabetic kidney disease (DKD), hypertensive nephropathy (HN), lupus nephritis (LN), minimal change disease (MCD), membranous nephropathy (MN), and mesangial proliferative glomerulonephritis (MsPGN). Nuclei stained with DAPI (4',6'-diamidino-2-phenylindole) are shown in blue. Scale bar, 50 μm. (B) Dectin-1 is highly enriched in macrophages, identified from an independent public data: GSE199711. (C) Representative dual-immunofluorescence staining of Dectin-1 and CD68 in the fibrotic renal tissues of patients with CKD. Scale bar, 50 μm. (D) Representative photomicrographs of Hematoxylin and eosin (H&E) staining, Periodic Acid-Schiff (PAS) staining, Periodic-acid silver methenamine (PASM) staining, and Masson's trichrome staining in IgA nephropathy (IgAN). Scale bar, 50 μm. (E) Representative photomicrographs of Dectin-1 immunofluorescence staining in IgAN (n = 42 for each group). Scale bar, 50 μm. (F) Quantification of the Dectin-1^+^ cells in (E). (G) The correlation between the number of Dectin-1^+^ cells and serum creatinine (Scr), blood urea nitrogen (BUN), urinary protein, fibrosis, serum albumin, and estimated glomerular filtration rate (eGFR) in IgAN patients (n = 84). Data are presented as mean ± SD. ****P < 0.0001.

We first examined Dectin-1 expression in renal biopsy samples from CKD patients. Immunofluorescence staining revealed a significant upregulation of Dectin-1 in fibrotic renal tissues (Figure 1A). Then, we sought to identify the specific cells primarily responsible for Dectin-1 expression in renal fibrosis. Analysis of an independent public dataset (GEO: GSE199711) revealed that Dectin-1 is predominantly expressed in renal macrophages (Figure 1B). Double-immunofluorescence staining for Dectin-1 and CD68 further confirmed that Dectin-1 expression lies mainly with macrophages in the fibrotic renal tissues of CKD patients (Figure 1C). Next, to further investigate the association between Dectin-1 expression and CKD progression, we enrolled 84 IgA nephropathy (IgAN) patients and categorized them into T0 and T1 groups based on tubular atrophy and interstitial fibrosis, following the IgAN Oxford classification (Figure 1D, Supplementary Table S1). Immunofluorescence staining showed a significantly higher number of Dectin-1⁺ cells in T1 group kidneys compared to the T0 group (Figure 1E and F).

To understand the relationship between Dectin-1 expression and progressive renal injury, we divided IgAN patients into two groups based on the median number of Dectin-1⁺ cells. As shown in Table 1, compared to those with lower Dectin-1^+^ cell counts, patients with higher Dectin-1^+^ cell counts developed more severe renal functional injury including significantly elevated levels of serum creatinine, blood urea nitrogen, urinary protein, urinary protein-to-creatinine ratio, but significantly declined in eGFR and serum albumin levels, and developed much more severe renal fibrosis. All these changes were further confirmed by correlation analysis (Figure 1G), revealing that Dectin-1 overexpression may contribute to the development of CKD, particularly progressive renal fibrosis. Univariable regression analysis identified the number of Dectin-1⁺ cells, serum creatinine, blood urea nitrogen, serum albumin, urinary albumin-to-creatinine ratio, eGFR, and hypertension as factors associated with renal fibrosis in IgAN patients. Multivariable analysis further highlighted the number of Dectin-1⁺ cells as a significant risk factor for renal fibrosis (Table 2).

By using public RNA-seq datasets of CKD patients from the Gene Expression Omnibus (GEO: GSE137570), we found that *CLEC7A* expression was significantly elevated in CKD patients with more severe renal fibrosis and significantly associated with severe renal fibrosis and the decline in eGFR (Figure S1A and B). Additionally, patients with progressive CKD exhibited higher levels of *CLEC7A* expression compared to those with stable CKD (Figure S1C), suggesting that Dectin-1 may contribute to progressive CKD.

### Dectin-1 deficiency attenuates renal fibrosis

**Figure 2:**
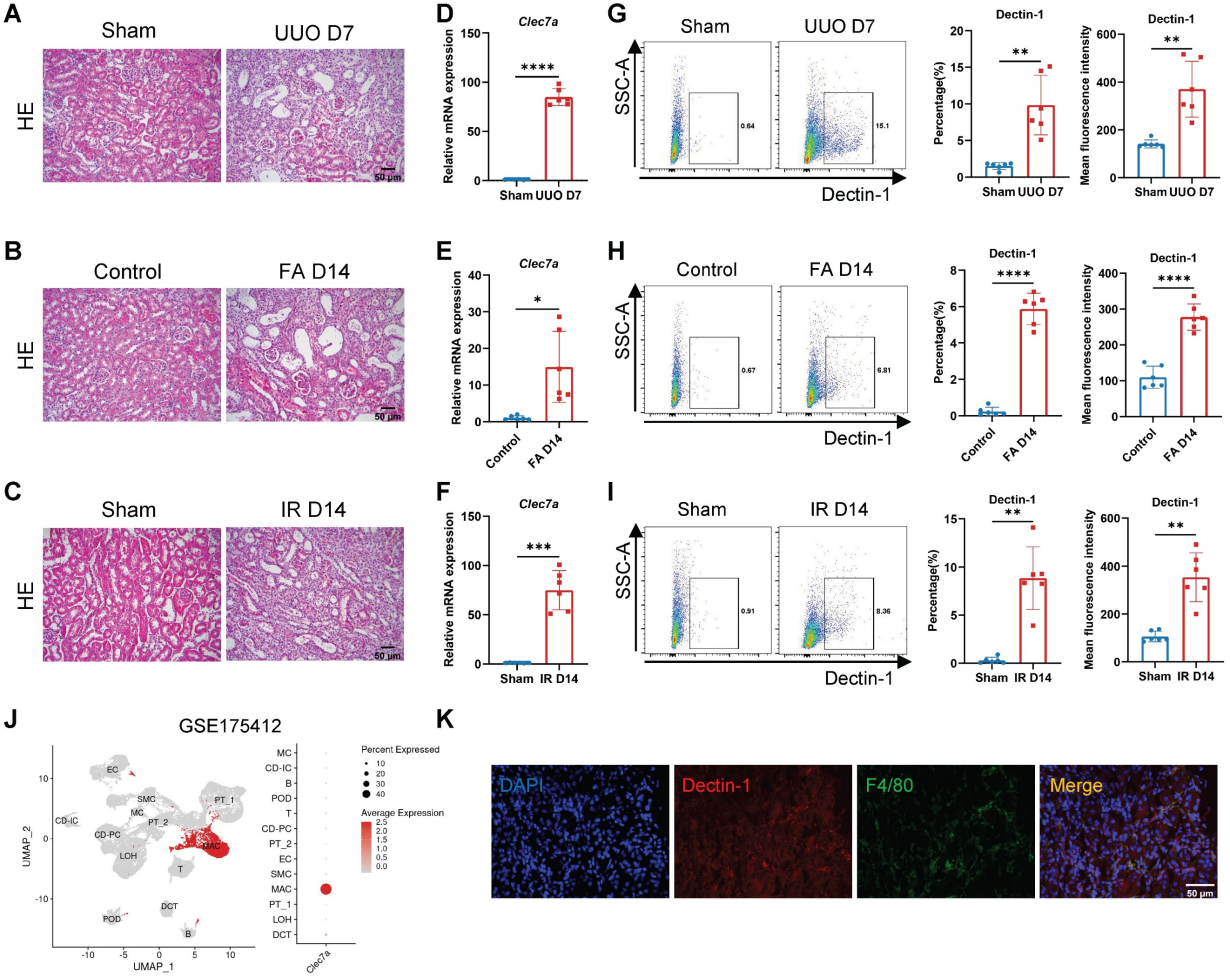
** Dectin-1 is markedly upregulated in mouse chronic kidney disease (CKD) models induced by unilateral ureteric obstruction (UUO), folic acid (FA), and ischemia-reperfusion (IR) and is presumably expressed by macrophages.** (A) Representative photomicrographs illustrating H&E staining in the kidney tissues of mice at day 7 after UUO or sham operation. Scale bar, 50 μm. (B) Representative photomicrographs illustrating H&E staining in the kidney tissues of mice at day 14 after FA induction. Scale bar, 50 μm. (C) Representative photomicrographs illustrating H&E staining in the kidney tissues of mice at day 14 after IR or sham operation. Scale bar, 50 μm. (D) Relative mRNA expression of *Clec7a* (Dectin-1) in the kidney tissues of mice at day 7 after UUO or sham operation (n = 6 for each group). (E) Relative mRNA expression of *Clec7a* (Dectin-1) in the kidney tissues of mice at day 14 after FA induction (n = 6 for each group). (F) Relative mRNA expression of *Clec7a* (Dectin-1) in the kidney tissues of mice at day 14 after IR or sham operation (n = 6 for each group). (G) Representative flow cytometric dot plots (left) and quantifications (2 parts in the right) illustrating the percentage of Dectin-1^+^ cells and its mean fluorescence intensity in the kidney tissues of mice at day 7 after UUO or sham operation (n=6 for each group). (H) Representative flow cytometric dot plots (left) and quantifications (2 parts in the right) illustrating the percentage of Dectin-1^+^ cells and its mean fluorescence intensity in the kidney tissues of mice at day 14 after FA induction (n=6 for each group). (I) Representative flow cytometric dot plots (left) and quantifications (2 parts in the right) illustrating the percentage of Dectin-1^+^ cells and its mean fluorescence intensity in the kidney tissues of mice at day 14 after IR or sham operation (n=6 for each group). (J) Dectin-1 is highly enriched in macrophages, identified from an independent public data: GSE175412. (K) Representative dual-immunofluorescence staining of Dectin-1 and F4/80 in the fibrotic kidney tissues of mice. Scale bar, 50 μm. Data are presented as mean ± SD. *P<0.05, **P<0.01, ***P<0.001, ****P < 0.0001.

We next examined the functional role of Dectin-1 in CKD with progressive renal fibrosis in UUO, FA, and IR-associated mouse models. Histology (H&E) detected a progressive renal fibrosis developed in all mouse models in wild-type (WT) mice induced by UUO, FA, and IR, which was associated with a marked upregulation of *Clec7a* as determined by real-time PCR at the mRNA level and by flow cytometry at the cellular levels (Figure 2A-I). Analysis of an independent public dataset (GEO: GSE175412) revealed that Dectin-1 is primarily expressed in renal macrophages (Figure 2J). Double-immunofluorescence staining of Dectin-1 and F4/80 further verified that Dectin-1 is predominantly expressed by macrophages in the fibrotic kidney tissues of mice (Figure 2K).

**Figure 3:**
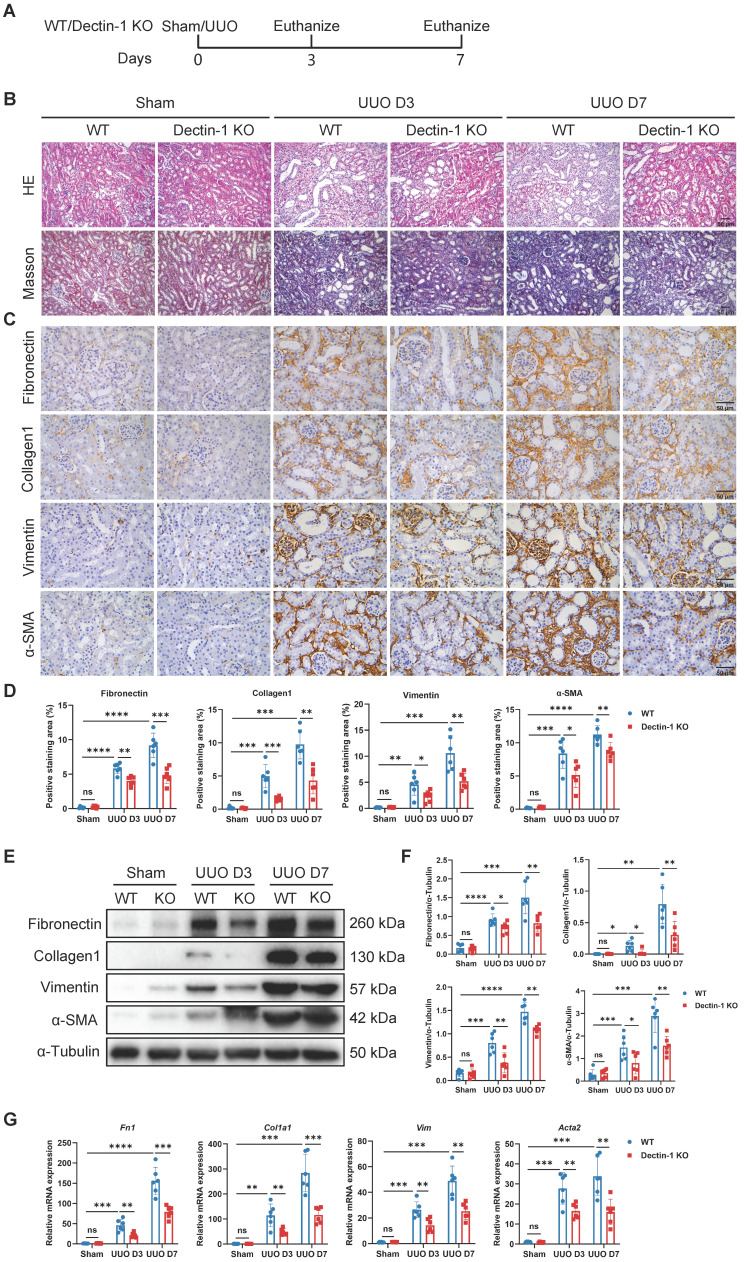
** Mice lacking Dectin-1 are protected from unilateral ureteric obstruction (UUO)-induced progressive renal fibrosis.** Wild-type (WT) and Dectin-1 knockout (KO) mice challenged to sham or UUO operation. (A) Experimental design of the treatment procedure. (B) Representative photomicrographs illustrating H&E and Masson's trichrome staining in kidney tissues. Scale bar, 50 μm. (C) Representative photomicrographs illustrating immunohistochemical staining for fibronectin, collagen1, vimentin, and α-SMA in kidney tissues (n = 6 for each group). Scale bar, 50 μm. (D) Quantification of the positive staining area (%) in (C). (E) Representative western blot analysis of fibronectin, collagen1, vimentin, and α-SMA protein in the kidney tissues. α-Tubulin was used as loading control (n = 6 for each group). (F) Densitometric quantification of blots in (E). (G) Relative mRNA expression of *Fn1*, *Col1a1*, *Vim*, and *Acta2* in kidney tissues (n = 6 for each group). Data are presented as mean ± SD. *P < 0.05, **P < 0.01, ***P < 0.001, ****P < 0.0001.

To assess the impact of Dectin-1 loss on renal fibrosis, we used Dectin-1 KO mice, which exhibit undetectable Dectin-1 protein levels in kidney tissues (Figure S2). Both Dectin-1 KO and WT mice underwent UUO and IR surgeries (Figure 3A). Histological analysis with H&E and Masson's trichrome staining revealed significant increases in interstitial extracellular matrix (ECM) deposition in UUO mice on days 3 and 7, compared to sham-operated controls. In contrast, renal interstitial fibrosis was markedly reduced in Dectin-1 KO mice compared to WT mice (Figure 3B). Immunohistochemistry further demonstrated that the elevated expression of fibronectin (an ECM glycoprotein), collagen I, vimentin (a mesenchymal marker), and α-SMA (a myofibroblast marker) in fibrotic kidneys was substantially reduced in Dectin-1 KO mice compared to WT mice (Figure 3C, D). Both protein and mRNA levels of these fibrotic markers were significantly higher in fibrotic kidneys but markedly lower in Dectin-1 KO mice compared to WT controls (Figure 3E-G). Similarly, Dectin-1 deficiency also attenuated IR-induced renal fibrosis (Figure S3).

### Dectin-1 expressed on bone marrow (BM)-derived cells mediates renal fibrosis

**Figure 4:**
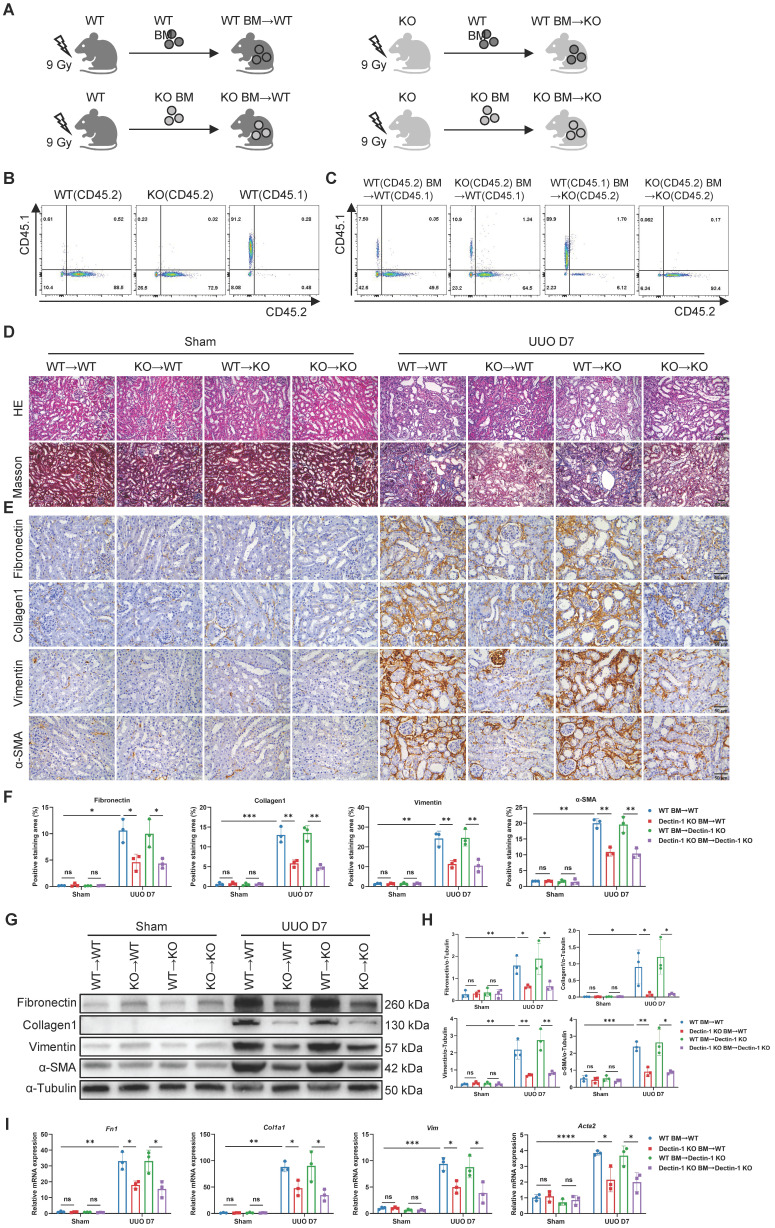
** Deletion of bone marrow (BM)-derived Dectin-1 inhibits unilateral ureteric obstruction (UUO)-induced renal fibrosis.** (A) Experimental design of the treatment procedure. (B) Flow cytometric analysis showing the genetic background of CD45 alleles in the wild-type (WT) and Dectin-1 knockout (KO) mice. (C) Successful BM transplantations (BMTs) were confirmed by flow cytometric analysis of peripheral blood cells. (D-I) After successful BMT, mice from 4 groups (WT BM→WT, Dectin-1 KO BM→WT, WT BM→ Dectin-1 KO, and Dectin-1 KO BM→ Dectin-1 KO) were subjected to sham or UUO operation. (D) Representative photomicrographs illustrating H&E and Masson's trichrome staining in kidney tissues. Scale bar, 50 μm. (E) Representative photomicrographs illustrating immunohistochemical staining for fibronectin, collagen1, vimentin, and α-SMA in kidney tissues (n=3 for each group). Scale bar, 50 μm. (F) Quantification of the positive staining area (%) in (E). (G) Representative western blot analysis of fibronectin, collagen1, vimentin, and α-SMA protein in the kidney tissues. α-Tubulin was used as loading control (n = 3 for each group). (H) Densitometric quantification of blots in (G). (I) Relative mRNA expression of *Fn1*, *Col1a1*, *Vim*, and *Acta2* in kidney tissues (n = 3 for each group). Data are presented as mean ± SD. *P < 0.05, **P < 0.01, ***P < 0.001, ****P < 0.0001.

To further assess whether Dectin-1 expression in renal parenchymal cells or bone marrow (BM)-derived immune cells contributes to renal fibrosis, we performed BM transplants between Dectin-1 KO and WT mice (Figure 4A). Since Dectin-1 KO mice have a C57B/6 background and express the CD45.2 allele, we used a WT C57B/6 substrain expressing the CD45.1 allele to facilitate tracking. Flow cytometry confirmed the CD45.1 expression in WT C57B/6-CD45.1 mice, while both WT C57B/6-CD45.2 and Dectin-1 KO mice expressed CD45.2 (Figure 4B). Four weeks post-BMT, we verified successful BM reconstitution in recipient mice via flow cytometry of peripheral blood cells (Figure 4C). These reconstituted mice then underwent UUO surgery.

Seven days after UUO, we evaluated the extent of renal fibrosis in the recipient mice. H&E and Masson's trichrome staining revealed significantly reduced ECM accumulation in the interstitial areas of kidneys from WT mice transplanted with Dectin-1 KO BM-derived cells (Dectin-1 KO BM→WT) compared to those transplanted with WT BM (WT BM→WT). In contrast, WT BM transplantation into Dectin-1 KO mice (WT BM→Dectin-1 KO) resulted in more severe interstitial fibrosis than when Dectin-1 KO BM-derived cells were transplanted into Dectin-1 KO mice (Dectin-1 KO BM→Dectin-1 KO) (Figure 4D). Immunohistochemical analysis confirmed these findings, showing decreased expression of fibronectin, collagen I, vimentin, and α-SMA in the kidneys of the Dectin-1 KO BM→WT group compared to the WT BM→WT group. Conversely, fibrotic protein deposition was markedly higher in the kidneys of WT BM→Dectin-1 KO mice, suggesting that the extent of renal fibrosis is influenced by the Dectin-1 genotype of the BM-derived cells (Figure 4E, F). Furthermore, both protein and mRNA levels of these fibrotic markers were significantly reduced in the kidneys of mice transplanted with Dectin-1 KO BM-derived cells compared to those receiving WT BM (Figure 4G-I). These results indicate that Dectin-1 expressed on BM-derived cells, rather than renal parenchymal cells, plays a crucial role in the development of renal fibrosis.

### Dectin-1 expressed by macrophages mediates renal fibrosis

**Figure 5:**
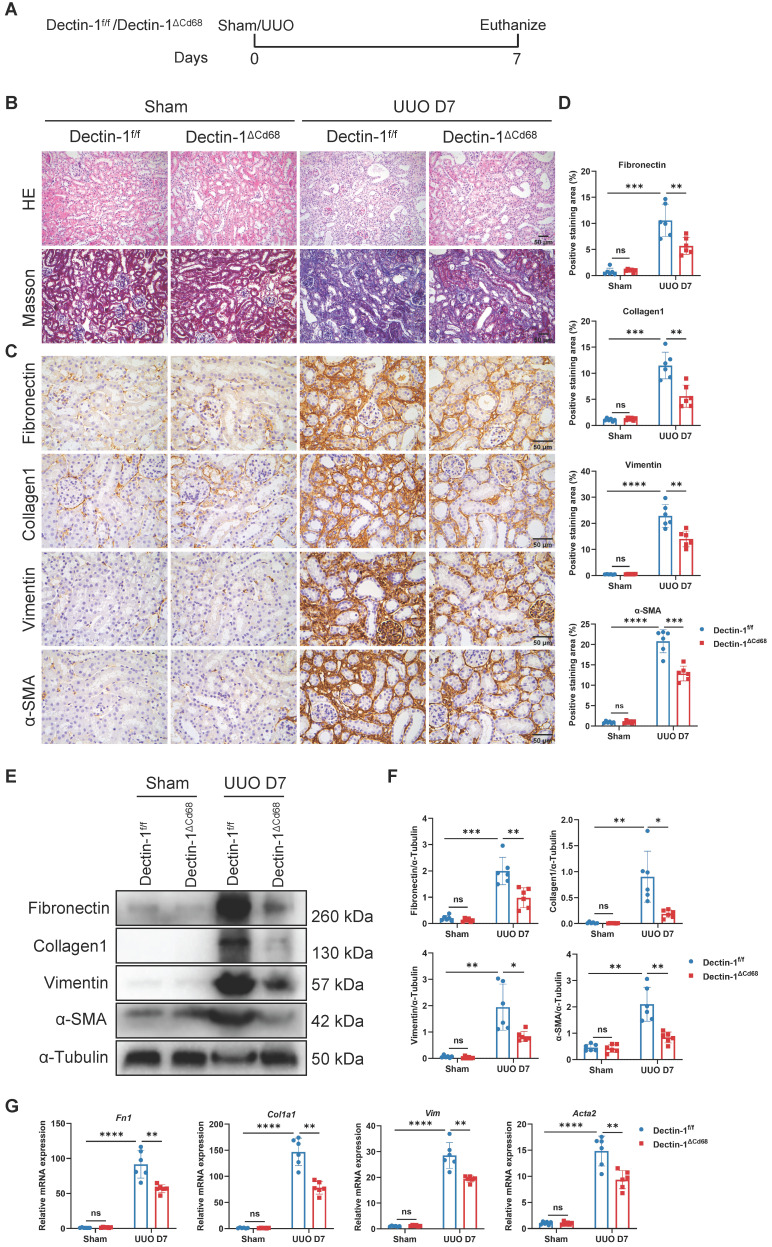
** Macrophage-specific deletion of Dectin-1 protects against unilateral ureteric obstruction (UUO)-induced renal fibrosis. Dectin-1^f/f^ and Dectin-1^ΔCd68^ mice challenged to sham or UUO operation.** (A) Experimental design of the treatment procedure. (B) Representative photomicrographs illustrating H&E and Masson's trichrome staining in kidney tissues. Scale bar, 50 μm. (C) Representative photomicrographs illustrating immunohistochemical staining for fibronectin, collagen1, vimentin, and α-SMA in kidney tissues (n=6 for each group). Scale bar, 50 μm. (D) Quantification of the positive staining area (%) in (C). (E) Representative western blot analysis of fibronectin, collagen1, vimentin, and α-SMA protein in the kidney tissues. α-Tubulin was used as loading control (n = 6 for each group). (F) Densitometric quantification of blots in (E). (G) Relative mRNA expression of *Fn1*, *Col1a1*, *Vim*, and *Acta2* in kidney tissues (n = 6 for each group). Data are presented as mean ± SD. *P < 0.05, **P < 0.01, ***P < 0.001, ****P < 0.0001.

To investigate whether macrophage-expressed Dectin-1 mediates renal fibrosis, we generated Dectin-1^△Cd68^ mice, in which Dectin-1 is specifically deleted in macrophages. Littermates of Dectin-1^f/f^ mice served as controls. The mRNA levels of *Clec7a* in BMDMs were significantly downregulated in Dectin-1^△Cd68^ mice compared to Dectin-1^f/f^ mice (Figure S4). Both Dectin-1^△Cd68^ and Dectin-1^f/f^ mice then underwent UUO (Figure 5A). While Dectin-1^f/f^ mice developed substantial collagen deposition in the interstitial areas of the UUO kidneys, Dectin-1^△Cd68^ mice showed markedly less collagen accumulation, as observed in H&E and Masson's trichrome staining (Figure 5B). Immunohistochemistry and Western blot analyses revealed that macrophage-specific Dectin-1 deletion reduced the upregulation of fibronectin, collagen I, vimentin, and α-SMA in the UUO kidneys (Figure 5C-F). Furthermore, mRNA levels of fibrosis-related genes were elevated in the kidneys of Dectin-1^f/f^ mice after UUO but significantly lower in Dectin-1^△Cd68^ mice (Figure 5G). These findings suggest that deletion of Dectin-1 specifically in macrophages attenuates renal fibrosis.

### Dectin-1 deficiency suppresses macrophage accumulation and activation in fibrotic kidneys

**Figure 6:**
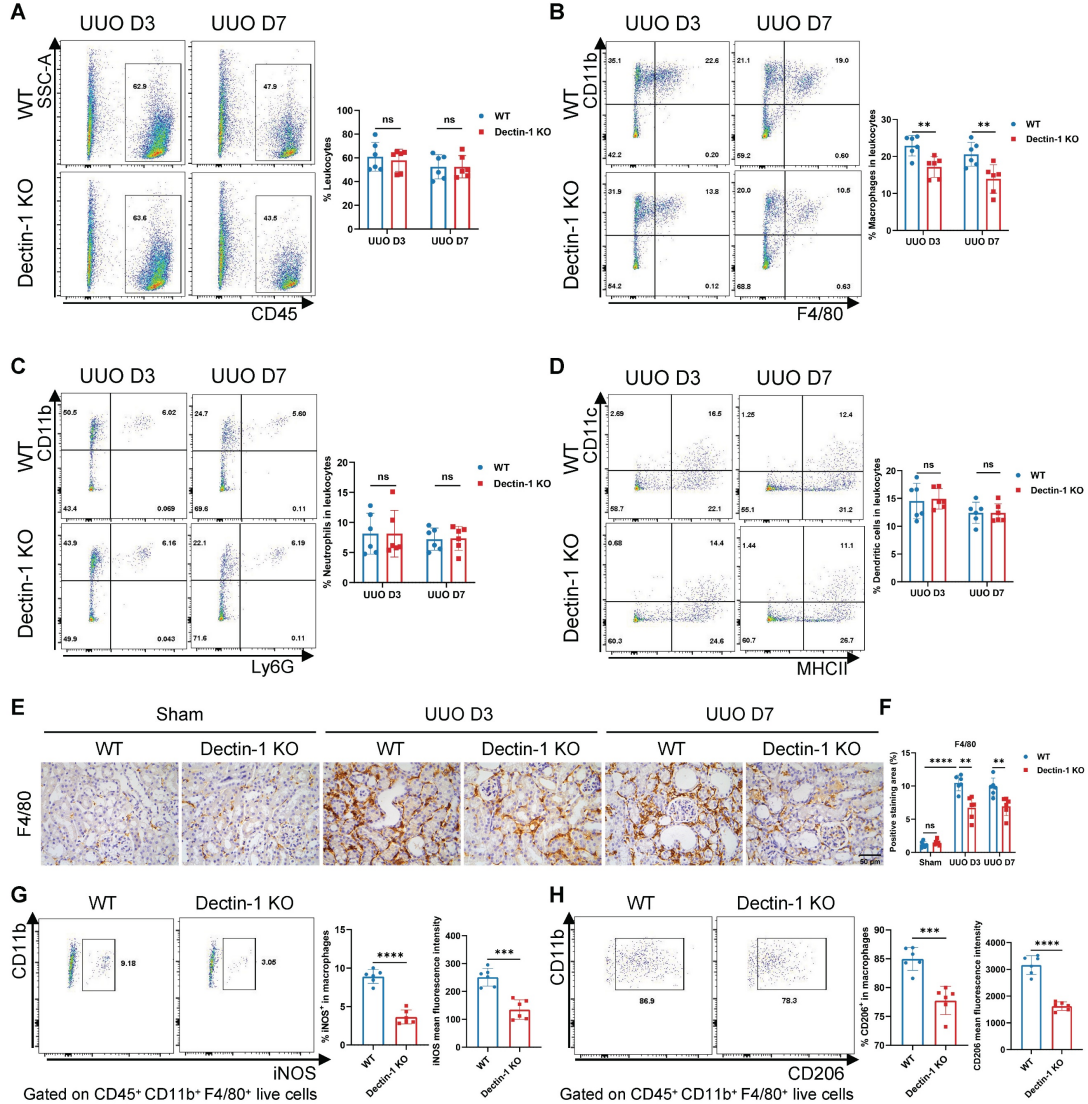
** Dectin-1 deficiency suppresses macrophage accumulation and activation in fibrotic kidneys.** Wild-type (WT) and Dectin-1 knockout (KO) mice subjected to sham or unilateral ureteric obstruction (UUO) operation. (A-D) Representative flow cytometric dot plots (left) and quantification (right) showing percentages of leukocytes (CD45^+^ live cells), macrophages (CD45^+^ CD11b^+^ F4/80^+^ live cells), neutrophils (CD45^+^ CD11b^+^ Ly6G^+^ live cells) and dendritic cells (CD45^+^ CD11c^+^ MHC II^+^ live cells) in kidneys (n=6 for each group). (E) Representative photomicrographs illustrating immunohistochemical staining for F4/80 in kidney tissues (n=6 for each group). Scale bar, 50 μm. (F) Quantification of the positive staining area (%) in (E). (G) Representative flow cytometric images illustrating the expression of iNOS^+^ in CD45^ +^ CD11b^ +^ F4/80^ +^ live cells (left) and the percentage of iNOS^+^ cells in macrophages and its mean fluorescence intensity levels (2 parts in the right) in kidney tissues 7 days after UUO (n=6 for each group). (H) Representative flow cytometric images illustrating the expression of CD206^+^ in CD45^ +^ CD11b^ +^ F4/80^ +^ live cells (left) and the percentage of CD206^+^ cells in macrophages and its mean fluorescence intensity levels (2 parts in the right) in kidney tissues 7 days after UUO (n=6 for each group). Data are presented as mean ± SD. **P < 0.01, ***P < 0.001, ****P < 0.0001.

We next examined whether Dectin-1 knockout influences inflammatory cell accumulation in renal fibrosis. Using flow cytometry, we analyzed WT and Dectin-1 KO mice following the UUO procedure, employing the gating strategy shown in Figure S5. Our results showed that Dectin-1 KO did not affect the percentage of CD45^+^ leukocytes among live cells or alter the proportions of CD11b^+^ Ly6G^+^ neutrophils and CD11c^+^ MHC II^+^ dendritic cells within the leukocyte population. However, Dectin-1 KO mice exhibited a significant reduction in CD11b^+^ F4/80^+^ macrophages in fibrotic kidneys compared to WT mice (Figure 6A-D). Immunohistochemistry further confirmed a marked decrease in F4/80^+^ macrophages in the UUO kidneys of Dectin-1 KO mice (Figure 6E, F). Notably, the proportions of macrophages, neutrophils, and dendritic cells in the spleen remained unchanged between WT and Dectin-1 KO mice (Figure S6). Moreover, on day 7 post-UUO induction, Dectin-1 KO mice exhibited a significant reduction in both the fraction of iNOS^+^ macrophages (M1 phenotype) and their mean fluorescence intensity compared to WT mice (Figure 6G). Similarly, the percentage of CD206^+^ macrophages (M2 phenotype) and their mean fluorescence intensity were significantly lower in Dectin-1 KO mice than in WT littermates (Figure 6H). Taken together, these data indicate that Dectin-1 plays a crucial role in macrophage accumulation and activation during renal fibrosis.

### Dectin-1 deletion reduces macrophage chemotaxis via the CCL2-CCR2 axis

**Figure 7:**
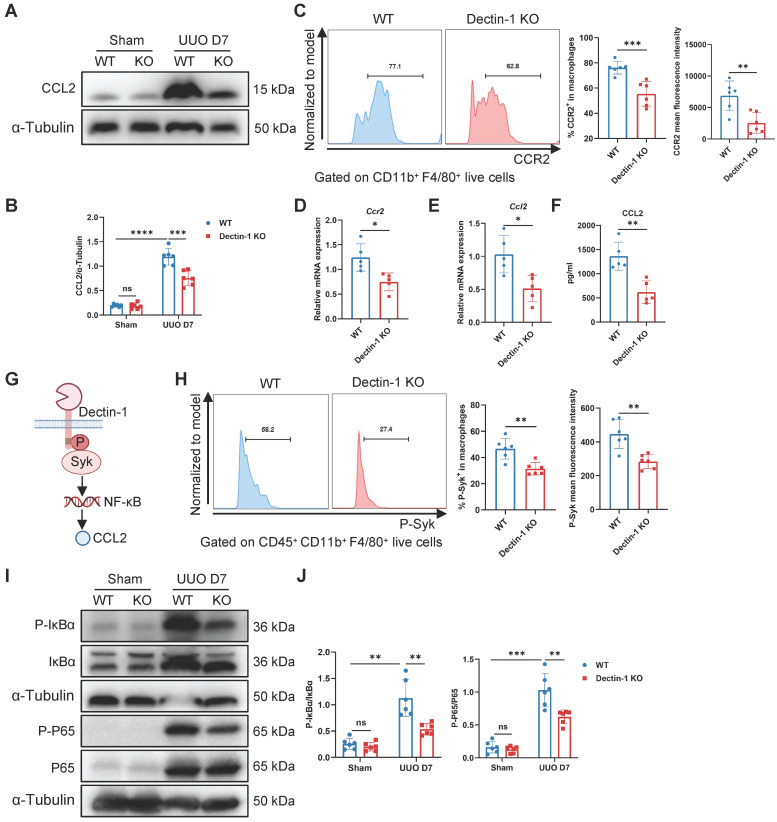
** Dectin-1 deletion reduces macrophage chemotaxis through CCL2 (C-C motif chemokine ligand 2)-CCR2 (C-C motif chemokine receptor 2) axis.** Wild-type (WT) and Dectin-1 knockout (KO) mice subjected to unilateral ureteric obstruction (UUO) operation. (A) Representative western blot analysis of CCL2 protein in the kidney tissues. α-Tubulin was used as loading control (n = 6 for each group). (B) Densitometric quantification of blots in (A). (C) Representative flow cytometric images illustrating the expression of CCR2^+^ in CD11b^+^ F4/80^+^ live cells (left) and the percentage of CCR2^+^ cells in macrophages and its mean fluorescence intensity levels (2 parts in the right) in kidneys (n=6 for each group). (D) Relative mRNA expression of *Ccr2* in sorted macrophages from fibrotic kidneys (n = 5 for each group). (E) Relative mRNA expression of *Ccl2* in sorted macrophages from fibrotic kidneys (n = 5 for each group). (F) The concentrations of CCL2 detected by ELISA in supernatant from sorted macrophages of fibrotic kidneys (n = 5 for each group). (G) Schematic of Dectin-1 signaling. (H) Representative flow cytometric images illustrating the expression of phospho (p-) spleen tyrosine kinase (Syk)^+^ in CD45^+^ CD11b^+^ F4/80^+^ live cells (left) and the percentage of p-Syk^+^ cells in macrophages and its mean fluorescence intensity levels (2 parts in the right) in kidneys (n=6 for each group). (I) Representative western blot analysis of p-IκBα, IκBα, p-p65, and p65 protein in the kidney tissues. α-Tubulin was used as loading control (n = 6 for each group). (J) Densitometric quantification of blots in (I). Data are presented as mean ± SD. *P < 0.05, **P < 0.01, ***P < 0.001, ****P < 0.0001.

To determine whether impaired macrophage proliferation contributes to the reduced macrophage numbers, we analyzed Ki-67^+^ macrophages by flow cytometry. This analysis revealed no significant differences in macrophage proliferation between the UUO kidneys of Dectin-1 KO and WT mice (Figure S7A). We then assessed the expression of receptors involved in macrophage chemotaxis in the UUO kidneys. Notably, Dectin-1 KO mice showed decreased mRNA levels of *Ccr2* and *Ccr7* compared to WT mice (Figure S7B). Furthermore, Dectin-1 deficiency significantly downregulated *Ccl2* mRNA expression, while *Ccl19* and *Ccl21* levels remained unchanged in the fibrotic kidneys of Dectin-1 KO mice (Figure S7C). These findings suggest that the CCL2-CCR2 axis may be involved in Dectin-1-mediated monocyte recruitment in fibrotic kidneys. Additionally, Western blot analysis confirmed that CCL2 expression was lower in the fibrotic kidneys of Dectin-1 KO mice compared to WT mice (Figure 7A, B). Flow cytometry further showed a significant reduction in CCR2^+^ macrophages and their mean fluorescence intensity in the fibrotic kidneys of Dectin-1 KO mice (Figure 7C). Moreover, F4/80^+^ macrophages isolated from fibrotic kidneys of mice subjected to UUO for 7 days exhibited a marked downregulation of *Ccr2* mRNA expression in the absence of Dectin-1 (Figure S7D, Figure 7D). Collectively, our data suggest that Dectin-1 regulates macrophage infiltration, with its effects mediated through the CCL2-CCR2 axis in the UUO model.

### Dectin-1 promotes macrophage expression of CCL2 via the Syk/NF-κB pathway

Macrophages reportedly exhibit the highest CCL2 expression in fibrotic kidneys 24. Western blot analysis revealed that CCL2 expression was reduced in fibrotic kidneys of Dectin-1^ΔCd68^ mice compared to Dectin-1^f/f^ mice (Figure S7E, F), suggesting that Dectin-1 deletion suppresses CCL2 expression in macrophages. Moreover, we isolated macrophages from the fibrotic kidneys of WT and Dectin-1 KO mice subjected to UUO for 7 days. These sorted macrophages were cultured for 24 hours, and ELISA was performed on the collected supernatants. The results revealed a significant reduction in CCL2 expression and secretion in macrophages from Dectin-1 KO mice compared to their WT littermates (Figure 7E, F). Next, we investigated whether Dectin-1 regulates CCL2 secretion through the canonical Syk/NF-κB pathway (Figure 7G). Flow cytometry showed a marked reduction in both the fraction of p-Syk^+^ macrophages and its mean fluorescence intensity in Dectin-1 KO mice at day 7 of UUO compared to WT mice (Figure 7H). Western blot analysis further demonstrated that while p-IκBα and p-p65 levels were significantly elevated in UUO kidneys, their expression was markedly reduced in Dectin-1 KO mice (Figure 7I, J). These findings suggest that Syk/NF-κB signaling plays a key role in Dectin-1-mediated macrophage infiltration.

To examine whether Dectin-1 deficiency affects CCL2 expression and secretion *in vitro*, we treated BMDMs from WT and Dectin-1 KO mice with the Dectin-1-specific agonist depleted zymosan (d-zymosan). In WT BMDMs, d-zymosan treatment significantly upregulated CCL2 expression and secretion, whereas this effect was substantially diminished in Dectin-1 KO BMDMs (Figure S8A, B). As expected, d-zymosan strongly induced p-Syk, p-IκBα, and p-p65 expression in BMDMs, but this activation was significantly suppressed in Dectin-1 KO BMDMs (Figure S8C, D). Similarly, d-zymosan-induced nuclear translocation of p65 was markedly reduced in Dectin-1 KO BMDMs compared to WT BMDMs (Figure S8E-H). Additionally, the Syk inhibitor R406 and the NF-κB inhibitor PDTC both suppressed CCL2 expression and secretion (Figure S8I, J), confirming that Dectin-1 regulates CCL2 through the Syk/NF-κB pathway.

### Dectin-1 contributes to the macrophage inflammatory response

Since NF-κB is a central regulator of inflammation 25, we investigated whether Dectin-1 modulates the macrophage inflammatory response. As expected, the mRNA expression of proinflammatory cytokines, including tumor necrosis factor-α (TNF-α), interleukin-6 (IL-6), and interleukin-1β (IL-1β), increased significantly after UUO but was markedly reduced in Dectin-1 KO mice compared to WT mice (Figure S9A). Similarly, sorted macrophages from the fibrotic kidneys of Dectin-1 KO mice exhibited lower expression and secretion of these cytokines than those from WT mice (Figure S9B, C).

To assess the functional impact of these macrophage-derived cytokines, we examined NGAL gene expression in HK-2 cells treated with macrophage-conditioned media. NGAL expression was significantly upregulated in HK-2 cells exposed to supernatants from sorted macrophages of WT UUO kidneys, whereas HK-2 cells showed reduced injury when cultured with supernatants from Dectin-1 KO renal macrophages (Figure S9D). *In vitro*, d-zymosan stimulation strongly induced TNF-α and IL-6 expression and secretion in BMDMs from WT mice, but this response was markedly attenuated in Dectin-1 KO BMDMs (Figure S9E, F). Similarly, treating WT BMDMs with d-zymosan in the presence of the Syk inhibitor R406 or the NF-κB inhibitor PDTC suppressed TNF-α and IL-6 levels (Figure S9G, H). Collectively, these findings indicate that Dectin-1 is involved in regulating macrophage-driven proinflammatory cytokine production.

### Dectin-1 deficiency reduces macrophage-to-myofibroblast transition (MMT) via the TGF-β/Smad pathway

**Figure 8:**
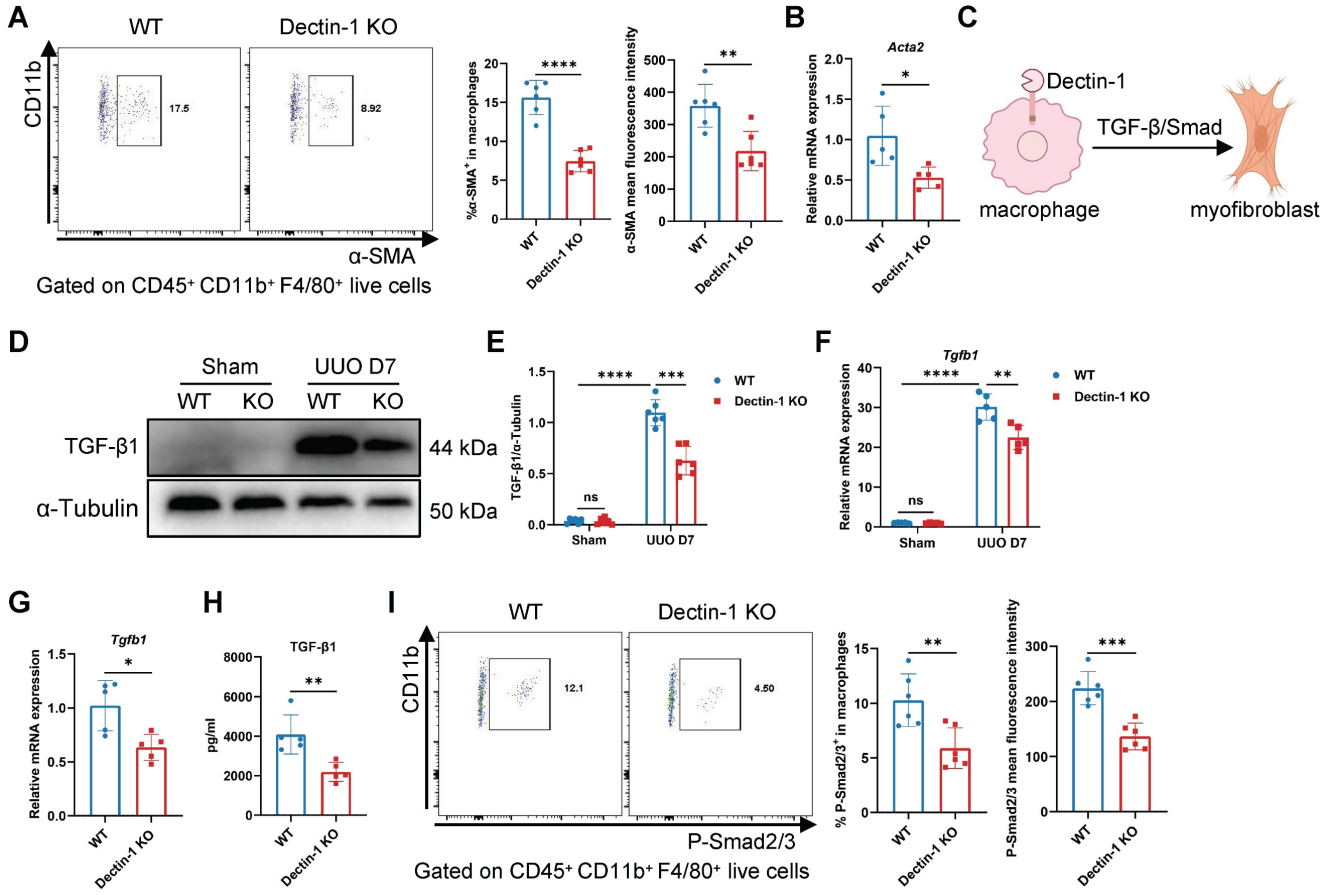
** Dectin-1 deficiency reduces macrophage-to-myofibroblast transition (MMT) through TGF-β/Smad pathway.** Wild-type (WT) and Dectin-1 knockout (KO) mice subjected to unilateral ureteric obstruction (UUO) operation. (A) Representative flow cytometric images illustrating the expression of α-SMA^+^ in CD45^+^ CD11b^+^ F4/80^+^ live cells (left) and the percentage of α-SMA^+^ cells in macrophages and its mean fluorescence intensity levels (2 parts in the right) in kidneys (n=6 for each group). (B) Relative mRNA expression of *Acta2* in sorted macrophages from fibrotic kidneys (n = 5 for each group). (C) Schematic of Dectin-1 induced MMT. (D) Representative western blot analysis of TGF-β1 protein in the kidney tissues. α-Tubulin was used as loading control (n = 6 for each group). (E) Densitometric quantification of blots in (D). (F) Relative mRNA expression of *Tgfb1* in kidney tissues (n = 5 for each group). (G) Relative mRNA expression of *Tgfb1* in sorted macrophages from fibrotic kidneys (n = 5 for each group). (H) The concentrations of TGF-β1 detected by ELISA in supernatant from sorted macrophages of fibrotic kidneys (n = 5 for each group). (I) Representative flow cytometric images illustrating the expression of p-Smad2/3^+^ in CD45^+^ CD11b^+^ F4/80^+^ live cells (left) and the percentage of p-Smad2/3^+^ cells in macrophages and its mean fluorescence intensity levels (2 parts in the right) in kidneys (n=6 for each group). Data are presented as mean ± SD. *P < 0.05, **P < 0.01, ***P < 0.001, ****P < 0.0001.

Macrophages infiltrating fibrotic kidneys can undergo MMT, differentiating into α-SMA^+^ myofibroblasts 26, 27. To determine whether macrophage-expressed Dectin-1 contributes to MMT, we analyzed α-SMA expression in renal macrophages. Flow cytometry revealed a significant reduction in both the number of α-SMA^+^ macrophages and their mean fluorescence intensity on day 7 of UUO in Dectin-1 KO mice compared to WT mice (Figure 8A), suggesting that Dectin-1 deficiency suppresses MMT. Consistently, sorted renal macrophages from Dectin-1 KO mice exhibited decreased *Acta2* mRNA expression (Figure 8B). TGF-β/Smad signaling is the central regulator of MMT 28, 29 (Figure 8C). Notably, the elevated TGF-β1 expression observed in fibrotic kidneys was significantly reduced in Dectin-1 KO mice compared to WT mice (Figure 8D-F). Similarly, sorted macrophages from UUO kidneys of Dectin-1 KO mice showed decreased TGF-β1 expression and secretion (Figure 8G, H). Moreover, both the proportion of p-Smad2/3^+^ macrophages and their mean fluorescence intensity were markedly lower in fibrotic kidneys of Dectin-1 KO mice relative to WT littermates (Figure 8I). These findings suggest that Dectin-1 promotes renal fibrosis by facilitating TGF-β/Smad-mediated MMT.

To further investigate Dectin-1's role in MMT, we cultured BMDMs with or without TGF-β1. In WT BMDMs, TGF-β1 significantly induced the expression of fibronectin, collagen 1, and α-SMA at both the mRNA and protein levels, whereas this response was abolished in Dectin-1 KO BMDMs (Figure S10A-E). Mechanistically, TGF-β1 stimulation led to a substantial increase in TGF-β1 production and Smad2/3 phosphorylation in WT BMDMs, confirming activation of the TGF-β/Smad pathway. However, these effects were notably suppressed in Dectin-1 KO BMDMs (Figure S10F-I). Taken together, these findings demonstrate that Dectin-1 promotes MMT by activating the TGF-β/Smad signaling pathway, contributing to renal fibrosis.

### Laminarin can ameliorate renal fibrosis

**Figure 9:**
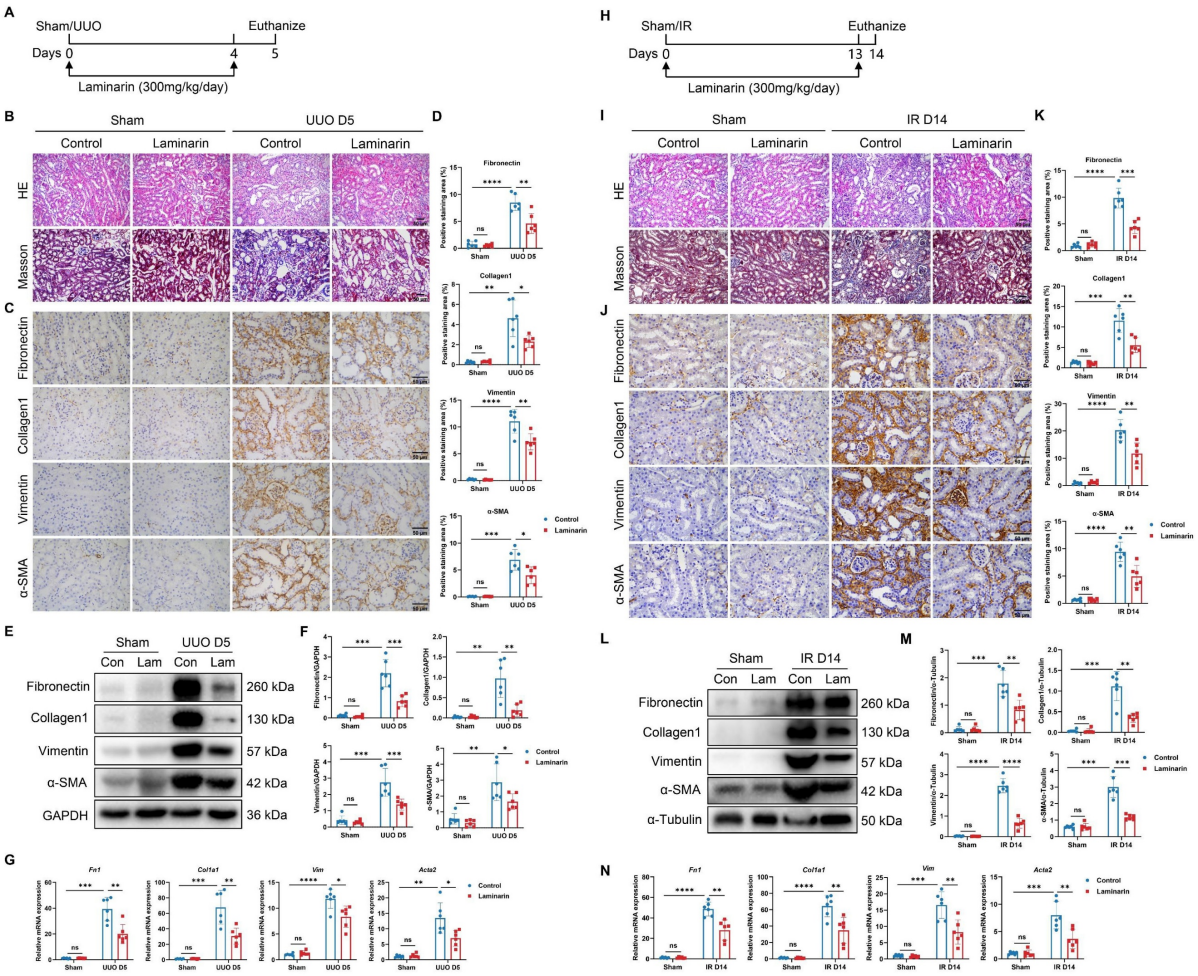
** Inhibition of Dectin-1 attenuates chronic kidney disease (CKD) progression induced by unilateral ureteric obstruction (UUO) and ischemia-reperfusion (IR) in mice.** (A-G) Laminarin was administered (300mg/kg/day) following sham or UUO surgery. (A) Experimental design of the treatment procedure. (B) Representative photomicrographs illustrating H&E and Masson's trichrome staining in kidney tissues. Scale bar, 50 μm. (C) Representative photomicrographs illustrating immunohistochemical staining for fibronectin, collagen1, vimentin, and α-SMA in kidney tissues (n=6 for each group). Scale bar, 50 μm. (D) Quantification of the positive staining area (%) in (C). (E) Representative western blot analysis of fibronectin, collagen1, vimentin, and α-SMA protein in the kidney tissues. GAPDH was used as loading control (n = 6 for each group). (F) Densitometric quantification of blots in (E). (G) Relative mRNA expression of *Fn1*, *Col1a1*, *Vim*, and *Acta2* in kidney tissues (n = 6 for each group). (H-N) Laminarin was administered (300mg/kg/day) following sham or IR surgery. (H) Experimental design of the treatment procedure. (I) Representative photomicrographs illustrating H&E and Masson's trichrome staining in kidney tissues. Scale bar, 50 μm. (J) Representative photomicrographs illustrating immunohistochemical staining for fibronectin, collagen1, vimentin, and α-SMA in kidney tissues (n=6 for each group). Scale bar, 50 μm. (K) Quantification of the positive staining area (%) in (J). (L) Representative western blot analysis of fibronectin, collagen1, vimentin, and α-SMA protein in the kidney tissues. α-Tubulin was used as loading control (n = 6 for each group). (M) Densitometric quantification of blots in (L). (N) Relative mRNA expression of *Fn1*, *Col1a1*, *Vim*, and *Acta2* in kidney tissues (n = 6 for each group). Data are presented as mean ± SD. *P < 0.05, **P < 0.01, ***P < 0.001, ****P < 0.0001.

We next investigated whether inhibiting Dectin-1 could mitigate renal fibrosis. Laminarin, a (1→3)-β-glucan derived from seaweed, competitively blocks agonist binding to Dectin-1 11. We administered laminarin (300 mg/kg/day) following UUO and IR surgeries (Figure 9A). Histological analysis with H&E and Masson's trichrome staining showed improved tubular integrity and reduced interstitial fibrosis in UUO mice treated with laminarin compared to untreated controls (Figure 9B). Immunohistochemistry further demonstrated that fibrotic marker expression—including fibronectin, collagen I, vimentin, and α-SMA—was significantly lower in laminarin-treated mice than in controls (Figure 9C, D). Western blot analysis confirmed reduced expression of these fibrotic proteins in UUO mice receiving laminarin (Figure 9E, F). Similarly, fibrosis-related mRNA levels, which increased following UUO surgery, were significantly lower in laminarin-treated mice compared to controls (Figure 9G). Consistently, laminarin also attenuated IR-induced renal fibrosis (Figure 9H-N).

### Dectin-1 is positively correlated with CCL2 and TGF-β1 in kidneys of CKD patients

**Figure 10:**
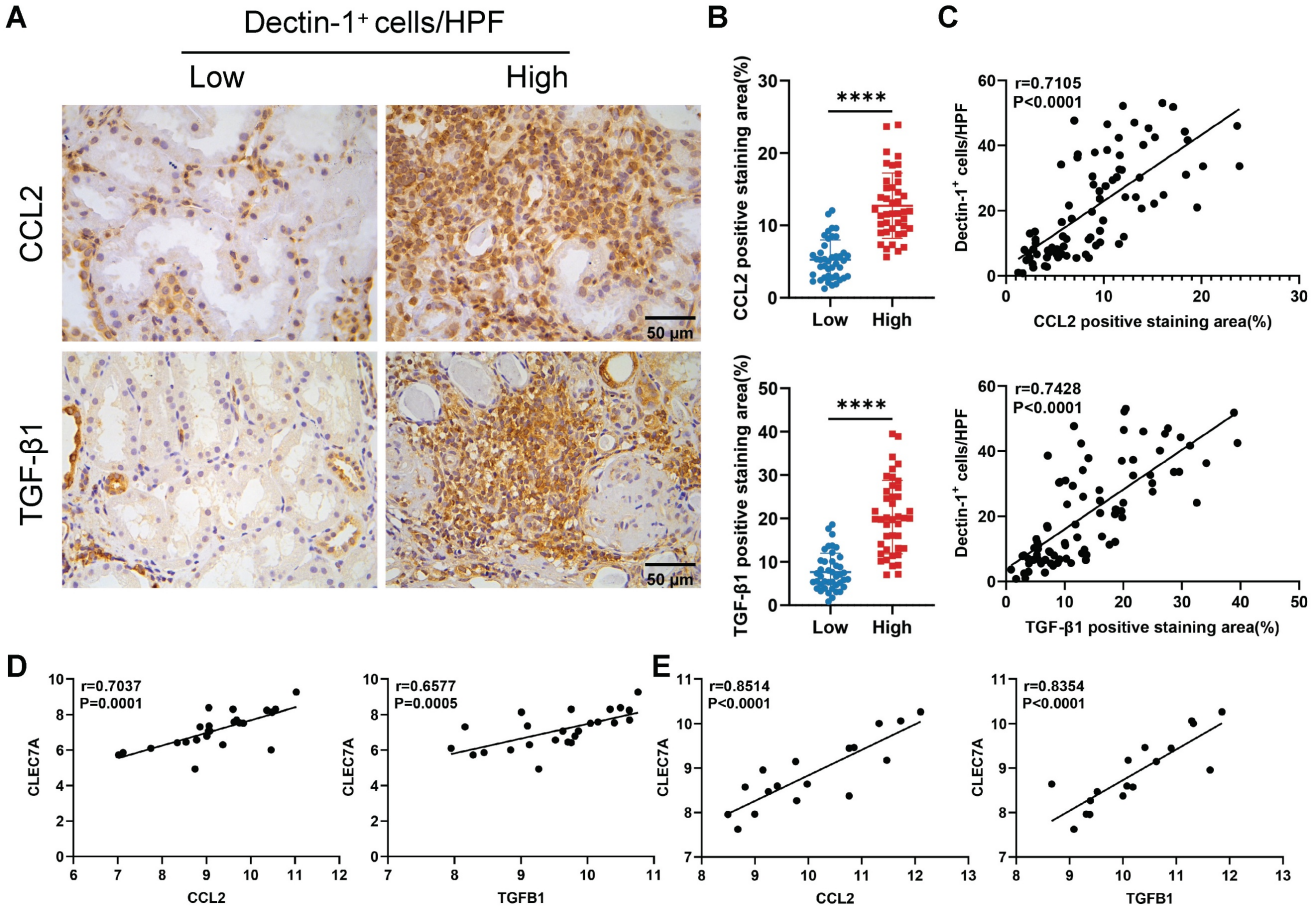
** Dectin-1 is positively correlated with CCL2 and TGF-β1 in the kidneys of patients with chronic kidney disease (CKD).** (A) Representative photomicrographs illustrating immunohistochemical staining for CCL2 and TGF-β1 in renal tissue of patients with IgA nephropathy (IgAN) (n = 42 for each group). Scale bar, 50 μm. (B) Quantification of the positive staining area (%) in (A). (C) The correlation of the number of Dectin-1^+^ cells with CCL2 and TGF-β1 expression in renal tissue of IgAN patients (n = 84). (D) The correlation of *CLEC7A* with CCL2 and TGFB1 in renal tissue of CKD patients in cohort 1 from GSE137570 (n = 24). (E) The correlation of *CLEC7A* with CCL2 and TGFB1 in renal tissue of CKD patients in cohort 2 from GSE137570 (n = 17). Data are presented as mean ± SD. ****P < 0.0001.

Finally, we assessed the clinical relevance of our experimental findings in CKD. As described earlier, we categorized 84 IgAN patients into two groups based on the median number of Dectin-1^+^ cells. Immunohistochemistry revealed significantly higher expression levels of CCL2 and TGF-β1 in the kidneys of patients with a greater number of Dectin-1^+^ cells compared to those with fewer Dectin-1^+^ cells (Figure 10A, B). Additionally, the number of Dectin-1^+^ cells correlated positively with both CCL2 and TGF-β1 expression (Figure 10C). Consistent with these results, a reanalysis of independent public data (GEO: GSE137570) confirmed that *CLEC7A* expression was positively correlated with CCL2 and TGFB1 in the kidneys of CKD patients (Figure 10D, E).

## Discussion

In this study, we found a significant increase of Dectin-1 expression in the fibrotic kidneys of CKD patients, and rise of Dectin-1 expression was correlated positively with renal fibrosis and negatively with kidney function. Regression analysis also suggested that Dectin-1 expression was an independent risk factor for renal fibrosis in IgAN. Genetic knockout of Dectin-1 significantly attenuated renal fibrosis induced by UUO or IR, primarily by suppressing macrophage infiltration via the Syk/NF-κB/CCL2-CCR2 axis and inhibiting MMT through the TGF-β/Smad pathway (Figure S11).

Dectin-1 has been shown to be highly expressed on myeloid-monocytic lineage cells, particularly macrophages 30. In a myocardial IR injury model, Fan et al. identified macrophages as the primary cell type expressing Dectin-1 31. In line with these findings, our BMTs strategy confirmed that Dectin-1 expressed on BM-derived cells mediates renal fibrosis. Furthermore, we demonstrated that Dectin-1 is predominantly expressed on macrophages in fibrotic kidneys. Notably, macrophage-specific Dectin-1 deletion effectively inhibited the progression of renal fibrosis, indicating that targeting Dectin-1 on macrophages could be a promising therapeutic strategy for the prevention of renal fibrosis.

Inflammation is a key component of renal fibrosis, and the fibrogenic niche promotes the infiltration of inflammatory cells 32. Our study uncovered that Dectin-1 deficiency decreased macrophage recruitment in fibrotic kidneys. These findings align with previous research on Dectin-1 in other systems. For example, Dectin-1 deletion prevented inflammatory macrophages infiltration in both heart allografts and Ang II-induced cardiac remodeling 22, 33. The CCL2-CCR2 signaling pathway is essential for the recruitment of BM-derived monocytes into injured kidneys 24, 34. Indeed, our results suggested that activation of CCL2-CCR2 signaling at least partly required Dectin-1. It is well established that Dectin-1 contains a hem-immunoreceptor tyrosine-based activation motif (ITAM) in its cytoplasmic tail, which triggers multiple downstream pathways, including the Raf-1 and Syk/NF-κB pathway 35, 36. Here, we showed that Dectin-1 deficiency reduced Syk phosphorylation and p65 nuclear translocation, resulting in decreased release of the chemokine CCL2, which recruits CCR2^+^ macrophages. Furthermore, the deletion of Dectin-1 diminished the production of inflammatory cytokines in macrophages, which have been identified as key drivers of fibrosis 37, 38.

During the progression of renal fibrosis, myofibroblasts are widely recognized as the main source of ECM production and deposition, which are primarily from the activation of resident fibroblasts and differentiation from BM-derived cells 26, 39. TGF-β/Smad signaling is the master regulator of MMT 28, 40. In the present study, we demonstrated that Dectin-1 promoted TGF-β1 secretion and Smad2/3 phosphorylation in macrophages, thereby facilitating MMT. Previous research has indicated that Dectin-1 could stimulate TGF-β1 expression in Ang II-induced macrophages through the Syk/p65 pathway 21, which are consistent with our findings.

In summary, our study identifies Dectin-1 as a critical mediator of renal fibrosis through dual mechanisms: (1) promoting macrophage infiltration via the Syk/NF-κB/CCL2-CCR2 axis, and (2) facilitating MMT through TGF-β/Smad activation. The correlation between Dectin-1 expression and disease severity in CKD patients, coupled with the protective effects of Dectin-1 deletion in experimental models, underscores its potential as a therapeutic target.

## Supplementary Material

Supplementary figures and tables.

## Figures and Tables

**Table 1:** Correlation of the number of Dectin-1^+^ cells with major clinicopathological parameters in IgAN patients.

	Number of Dectin-1^+^ cells		*P*
	Low (*n* = 42)	High (*n* = 42)	
Age (years)	37.55 ± 12.78	41.36 ± 14.17	0.1993
Sex (male)	23 (54.76%)	26 (61.90%)	0.5067
Serum creatinine (μmol/L)	90.29 ± 24.94	136.5 ± 44.4	< 0.0001
Blood urea nitrogen (mg/dL)	5.317 ± 1.29	7.567 ± 3.043	< 0.0001
Serum albumin (g/L)	44.21 ± 6.879	37.94 ± 4.041	< 0.0001
Urinary protein (g/d)	0.811 ± 0.7272	1.285 ± 0.8706	0.0111
Urinary albumin creatinine ratio	0.584 ± 0.5325	1.514 ± 1.401	0.0002
eGFR (ml/min/1.73m^2^)	87.15 ± 23.36	55.32 ± 19.74	< 0.0001
Diabetes mellitus (n, %)	2.48%	0	> 0.9999
Hypertension (n, %)	23.81%	50%	0.0129
Fibrosis (%)	8.095 ± 5.291	30.48 ± 8.89	< 0.0001

eGFR, estimated glomerular filtration rate.

**Table 2:** Logistic regression analysis of various clinicopathological factors and the number of Dectin-1^+^ cells for renal fibrosis in IgAN patients.

Factor	Univariable Analysis			Multivariable Analysis		
	HR	95% *CI*	*P*	HR	95% *CI*	*P*
Dectin-1^ +^ cells/HPF	1.587	1.244-2.025	< 0.0001	1.602	1.179-2.177	0.003
Scr (μmol/L)	1.052	1.029-1.076	< 0.0001	-	-	-
BUN (mg/dL)	1.881	1.320-2.680	< 0.0001	1.020	0.315-3.303	0.973
Serum albumin (g/L)	0.723	0.621-0.843	< 0.0001	0.972	0.612-1.543	0.903
Urinary protein (g/d)	1.733	0.973-3.088	0.062	-	-	-
UACR	3.505	1.677-7.323	0.001	0.641	0.096-4.279	0.646
eGFR (ml/min/1.73m2)	0.936	0.910-0.962	< 0.0001	0.984	0.907-1.067	0.695
Hypertension	0.236	0.090-0.615	0.003	0.662	0.041-10.73	0.771

HPF, high-power field; Scr, serum creatinine; BUN, blood urea nitrogen; UACR, urinary albumin creatinine ratio; eGFR, estimated glomerular filtration rate; HR, hazard ratio.
