# Supplementary material for: Dectin-1 is Pathogenic in Chronic Kidney Disease by Promoting Macrophage Infiltration and Transition to Myofibroblast
Source: Int J Biol Sci. 2025 Aug 16;21(12):5287–304. doi: 10.7150/ijbs.119129 (PMC12435479; doi:10.7150/ijbs.119129)
Supplement: Supplementary file 1 — Supplementary figures and tables. [file ijbsv21p5287s1.pdf]

Supplementary materials

Dectin-1 is Pathogenic in Chronic Kidney Disease by Promoting Macrophage Infiltration and Transition to Myofibroblast

Lingling Shen, Jingyi Li, Anqi Zhang, Sijing Yan, Wenxin Sha, Yucheng Wang, Shi Feng, Cuili Wang, Zhimin Chen, Hongfeng Huang, Bingjue Li, Pingping Ren, Suhan Zhou, Siqi Wu, Yanli Wang, Zhouji Shen, Song Rong, Hermann Haller, Hong Jiang, Jianghua Chen

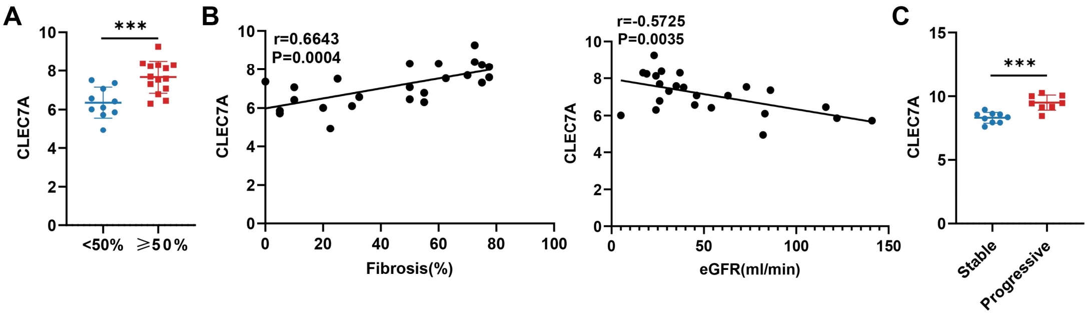

Supplementary Figure S1. High upregulation of *CLEC7A* and its correlation with pathological and clinical parameters in fibrotic kidneys of patients with chronic kidney disease (CKD). (A) Relative mRNA expression of *CLEC7A* in the renal tissue of CKD patients with renal fibrosis degree < 50% (n = 10) compared to those with renal fibrosis degree ≥ 50% (n = 14) in cohort 1 from GSE137570. (B) The correlation of the expression level of *CLEC7A* with the degree of renal fibrosis and estimated glomerular filtration rate (eGFR) in CKD patients in cohort 1 from GSE137570 (n = 24). (C) Relative mRNA expression of *CLEC7A* in the renal tissue of stable CKD patients (n = 9) compared to progressive CKD patients (n = 8) in cohort 2 from GSE137570. Data are presented as mean ± SD. ****P*<0.001.

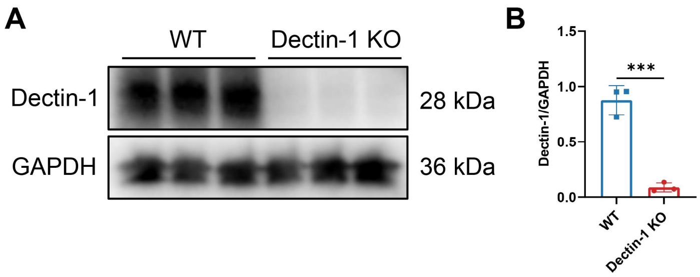

Supplementary Figure S2. Validation of Dectin-1 knockout (KO) mice. (A) Representative western blot analysis of Dectin-1 protein in the kidney tissues of wild-type (WT) and Dectin-1 KO mice. GAPDH was used as loading control (n = 3 for each group). (B) Densitometric quantification of blots in (A). Data are presented as mean ± SD. ****P*<0.001.

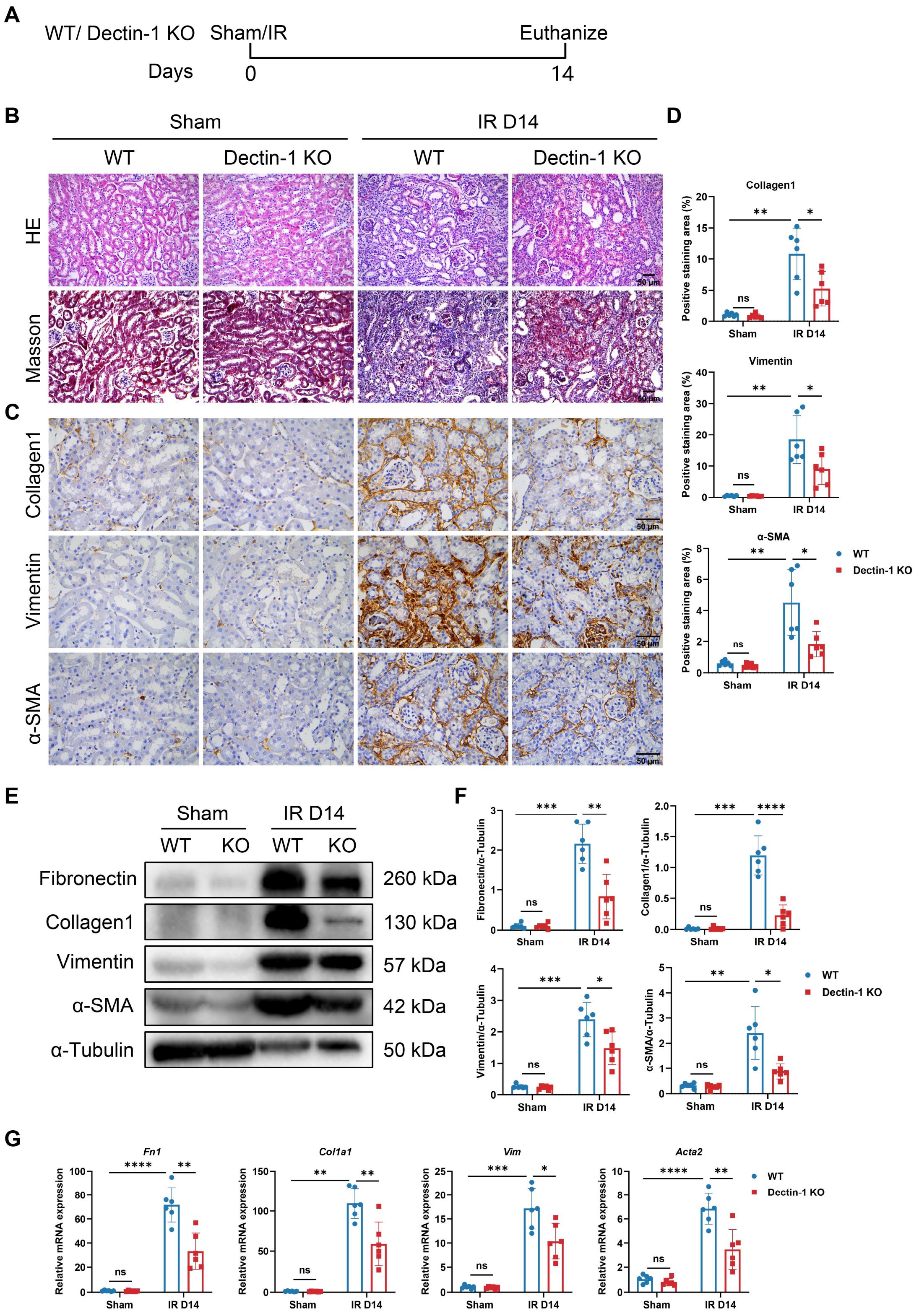

Supplementary Figure S3. Deletion of Dectin-1 protects against ischemia-reperfusion (IR)-induced renal fibrosis. Wild-type (WT) and Dectin-1 knockout (KO) mice challenged to sham or IR operation. (A) Experimental design of the treatment procedure. (B) Representative photomicrographs illustrating H&E and Masson's trichrome staining in kidney tissues. Scale bar, 50 μm. (C) Representative photomicrographs

illustrating immunohistochemical staining for collagen1, vimentin, and α-SMA in kidney tissues (n=6 for each group). Scale bar, 50 µm. (D) Quantification of the positive staining area (%) in (C). (E) Representative western blot analysis of fibronectin, collagen1, vimentin, and α-SMA protein in the kidney tissues. α-Tubulin was used as loading control (n = 6 for each group). (F) Densitometric quantification of blots in (E). (G) Relative mRNA expression of *Fn1*, *Col1a1*, *Vim*, and *Acta2* in kidney tissues (n = 6 for each group). Data are presented as mean ± SD. **P*<0.05, ***P*<0.01, ****P*<0.001, *****P* < 0.0001.

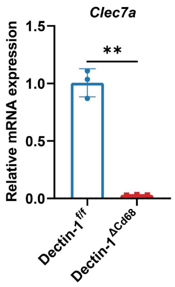

Supplementary Figure S4. Validation of Dectin-1$^{\Delta Cd68}$ mice. Relative mRNA expression of *Clec7a* in bone marrow-derived macrophages (BMDMs) from Dectin-1$^{f/f}$ and Dectin-1$^{\Delta Cd68}$ mice (n = 3 for each group). Data are presented as mean ± SD. ***P*<0.01.

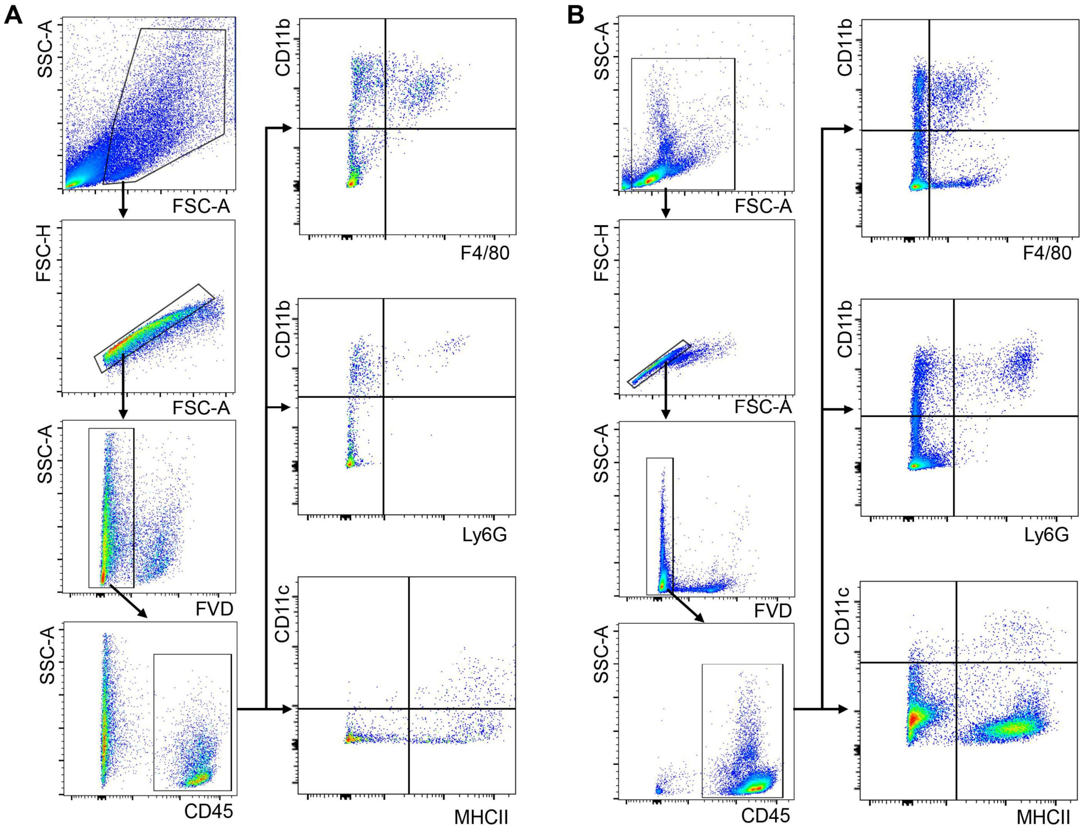

Supplementary Figure S5. Flow cytometry gating strategy. Representative flow cytometric dot plots determined leukocytes (CD45^{+} live cells), macrophages (CD45^{+}

CD11b$^+$ F4/80$^+$ live cells), neutrophils (CD45$^+$ CD11b$^+$ Ly6G$^+$ live cells) and dendritic cells (CD45$^+$ CD11c$^+$ MHC II$^+$ live cells). FVD- cells were gated as indicated for live cells. (A) Leukocytes, macrophages, neutrophils and dendritic cells examined in the kidneys. (B) Leukocytes, macrophages, neutrophils and dendritic cells examined in the spleens.

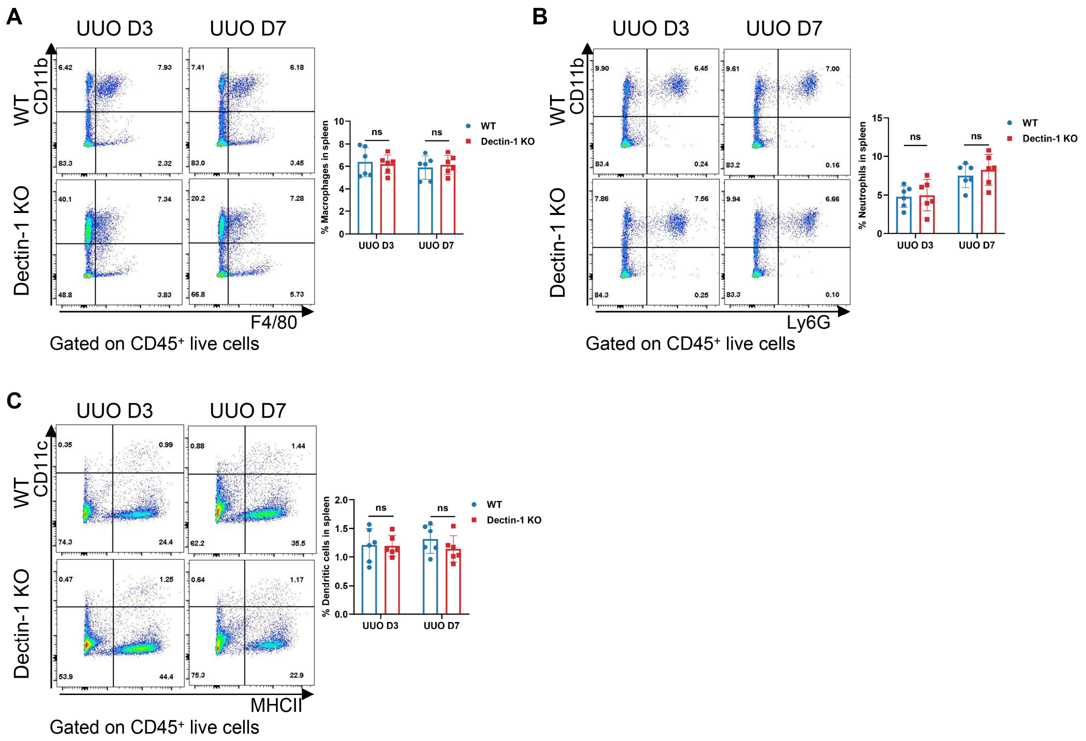

Supplementary Figure S6. Dectin-1 deletion has no effect on percentage of macrophages, neutrophils, and dendritic cells in the spleen. Wild-type (WT) and Dectin-1 knockout (D1 KO) mice subjected to unilateral ureteric obstruction (UUO) operation. (A-C) Representative flow cytometric dot plots (left) and quantification (right) showing percentages of macrophages (CD45$^+$ CD11b$^+$ F4/80$^+$ live cells), neutrophils (CD45$^+$ CD11b$^+$ Ly6G$^+$ live cells) and dendritic cells (CD45$^+$ CD11c$^+$ MHC II$^+$ live cells) in the spleen (n=6 for each group). Data are presented as mean ± SD.

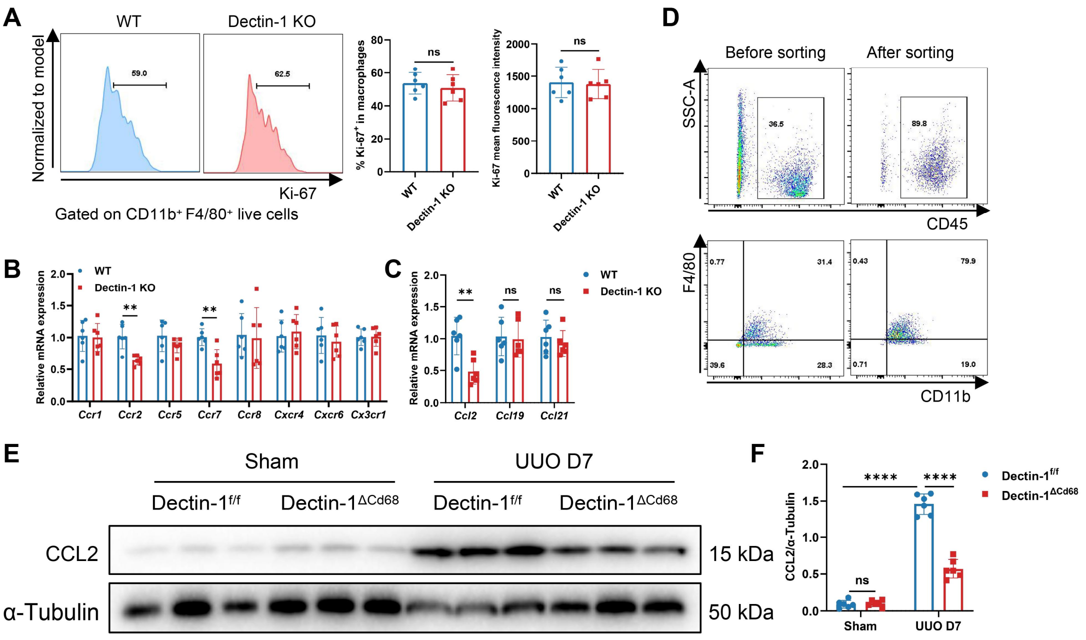

Supplementary Figure S7. Dectin-1 deletion reduces macrophage chemotaxis through CCL2 (C-C motif chemokine ligand 2)-CCR2 (C-C motif chemokine receptor 2) axis. (A-C) Wild-type (WT) and Dectin-1 knockout (KO) mice subjected to unilateral ureteric obstruction (UUO) operation. (A) Representative flow cytometric images illustrating the expression of Ki-67^{+} in CD11b^{+} F4/80^{+} live cells (left) and the percentage of Ki-67^{+} cells in macrophages and its mean fluorescence intensity levels (2 parts in the right) in kidneys (n=6 for each group). (B) Relative mRNA expression of *Ccr1*, *Ccr2*, *Ccr5*, *Ccr7*, *Ccr8*, *Cxcr4*, *Cxcr6*, and *Cx3cr1* in kidney tissues (n = 6 for each group). (C) Relative mRNA expression of *Ccl2*, *Ccl19*, and *Ccl21* in kidney tissues (n = 6 for each group). (D) Renal macrophages were isolated from the fibrotic kidneys. The purity of macrophages was determined by flow cytometry after sorting. (E) Representative western blot analysis of CCL2 protein in the kidney tissues of Dectin-1$^{f/f}$ and Dectin-1$^{\Delta Cd68}$ mice at day 7 after UUO or sham operation. α-Tubulin was used as loading control (n = 6 for each group). (F) Densitometric quantification of blots in (E). Data are presented as mean ± SD. $^{**}P<0.01$, $^{****}P < 0.0001$.

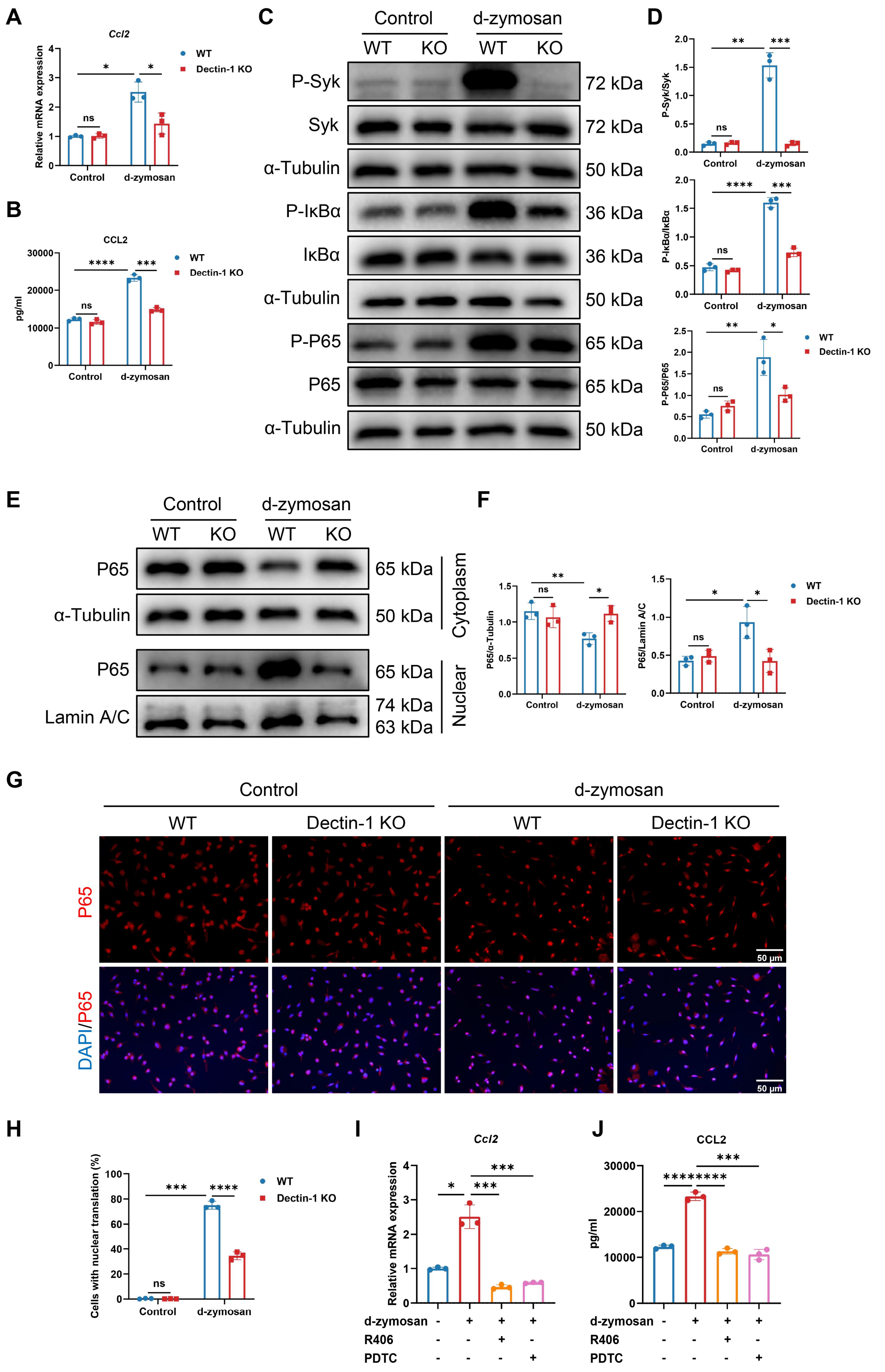

Supplementary Figure S8. Dectin-1 promotes the expression and secretion of CCL2 by macrophages via the Syk/NF-κB pathway. Bone marrow-derived macrophages

(BMDMs) from wild-type (WT) and Dectin-1 knockout (KO) mice with or without Dectin-1-specific agonist depleted zymosan (d-zymosan) (100 ug/mL) treatment. (A) Relative mRNA expression of *Ccl2* in BMDMs (n = 3 for each group). (B) The concentrations of CCL2 detected by ELISA in supernatant from BMDMs (n = 3 for each group). (C) Representative western blot analysis of p-Syk, Syk, p-IκBα, IκBα, p-p65, and p65 protein in BMDMs. α-Tubulin was used as loading control (n = 3 for each group). (D) Densitometric quantification of blots in (C). (E) Representative Western analysis of P65 in the nuclear extractions and cytoplasm of BMDMs. α-Tubulin and Lamin A/C were used as loading control (n = 3 for each group). (F) Densitometric quantification of blots in (E). (G) Representative immunofluorescence staining of P65 in BMDMs (n=3 for each group). Scale bar, 50 μm. (H) Quantification of p65 nuclear translocation in (G). (I-J) WT BMDMs were treated with Syk inhibitor (R406, 10 μM) or NF-κB inhibitor (PDTC, 20 μM) for 1 h, and then stimulated with d-zymosan (100 ug/mL). (I) Relative mRNA expression of *Ccl2* in BMDMs (n = 3 for each group). (J) The concentrations of CCL2 detected by ELISA in supernatant from BMDMs (n = 3 for each group). Data are presented as mean ± SD. **P*<0.05, ***P*<0.01, ****P*<0.001, *****P* < 0.0001.

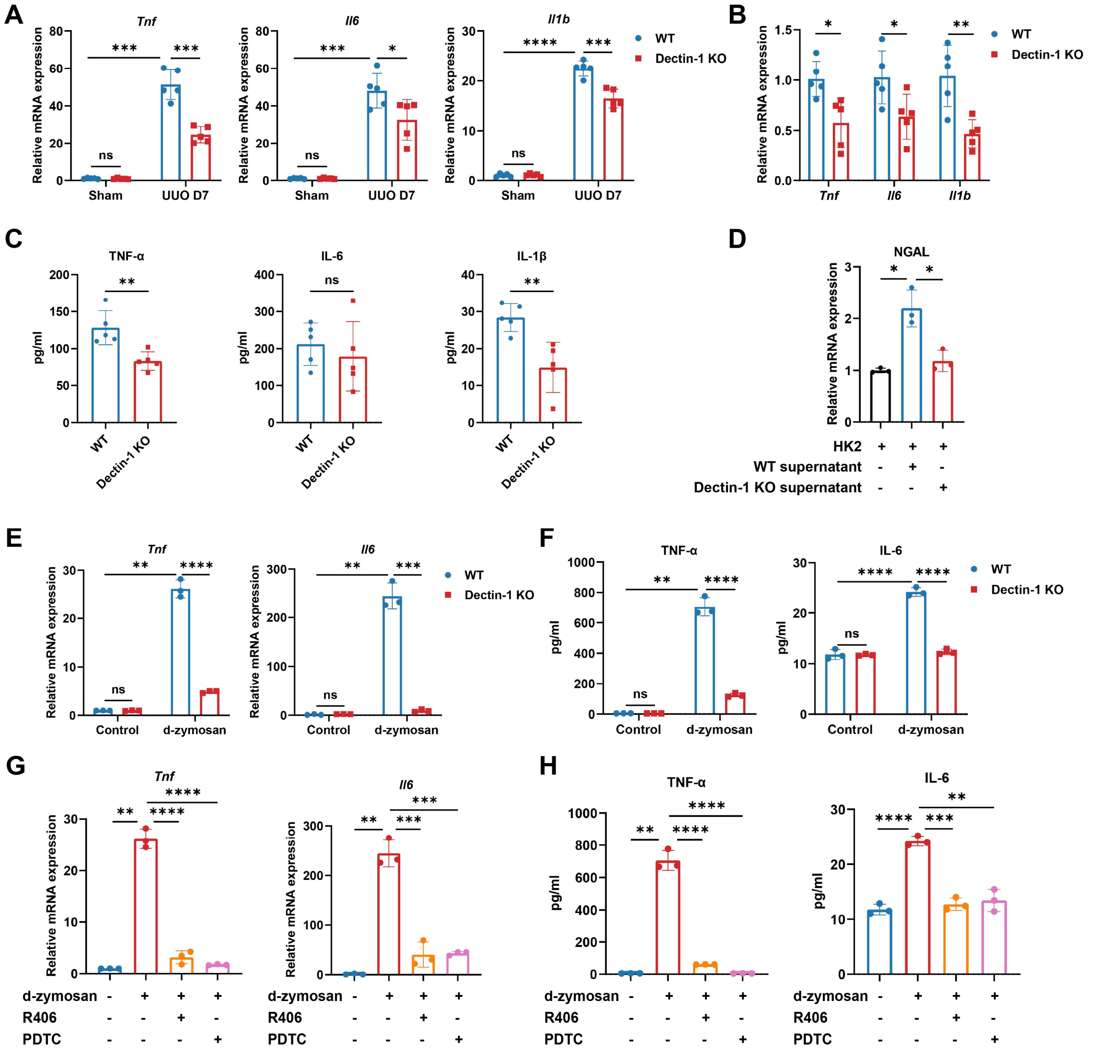

Supplementary Figure S9. Dectin-1 deficiency inhibits the expression and secretion of proinflammatory cytokines in macrophages. (A-D) Wild-type (WT) and Dectin-1 knockout (KO) mice subjected to unilateral ureteric obstruction (UUO) operation. (A) Relative mRNA expression of *Tnf*, *Il6*, and *Il1b* in kidney tissues (n = 5 for each group). (B) Relative mRNA expression of *Tnf*, *Il6*, and *Il1b* in sorted macrophages from fibrotic kidneys (n = 5 for each group). (C) The concentrations of tumor necrosis factor-α (TNF-α), interleukin-6 (IL-6), and interleukin-1β (IL-1β) detected by ELISA in supernatant from sorted macrophages of fibrotic kidneys (n = 5 for each group). (D) Relative mRNA expression of NGAL in HK-2 cells treated with conditioned medium from sorted macrophages of fibrotic kidneys (n = 3 for each group). (E-H) Bone marrow-derived macrophages (BMDMs) from WT and Dectin-1 KO mice with or without Dectin-1-specific agonist depleted zymosan (d-zymosan) (100 ug/mL) treatment. (E) Relative mRNA expression of *Tnf* and *Il6* in BMDMs (n = 3 for each group). (F) The concentrations of TNF-α and IL-6 detected by ELISA in supernatant from BMDMs (n = 3 for each group). (G-H) WT BMDMs were treated with Syk inhibitor (R406, 10 μM) or NF-κB inhibitor (PDTC, 20 μM) for 1 h, and then stimulated with d-zymosan (100 ug/mL). (G) Relative mRNA expression of *Tnf* and *Il6* in BMDMs (n = 3 for each group). (H) The concentrations of TNF-α and IL-6 detected by ELISA in supernatant from BMDMs (n = 3 for each group). Data are presented as mean ± SD. *P<0.05, **P<0.01, ***P<0.001, ****P < 0.0001.

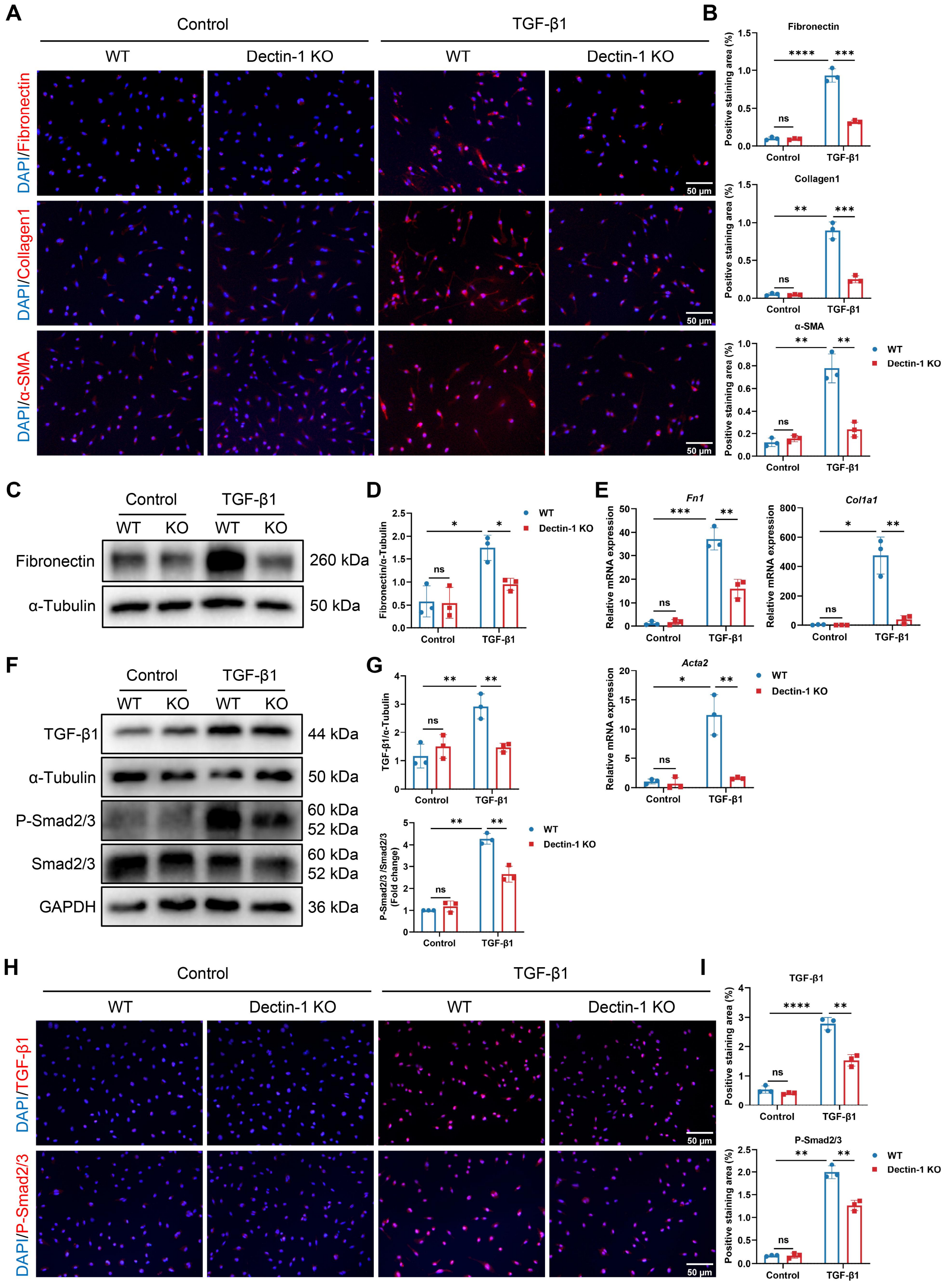

Supplementary Figure S10. Dectin-1 deletion inhibits TGF-β1-induced MMT by blocking TGF-β/Smad pathway. Bone marrow-derived macrophages (BMDMs) from wild-type (WT) and Dectin-1 knockout (KO) mice with or without TGF-β1 (5 ng/mL) treatment for 3 days. (A) Representative immunofluorescence staining of fibronectin, collagen1, and α-SMA in BMDMs (n=3 for each group). Scale bar, 50 μm. (B) Quantification of the positive staining area (%) in (A). (C) Representative Western analysis of fibronectin protein in BMDMs. α-Tubulin was used as loading control (n =

3 for each group). (D) Densitometric quantification of blots in (C). (E) Relative mRNA expression of *Fn1*, *Col1a1*, and *Acta2* in BMDMs (n = 3 for each group). (F) Representative Western analysis of TGF-β1, p-Smad2/3, and Smad2/3 protein in BMDMs. α-Tubulin or GAPDH was used as loading control (n = 3 for each group). (G) Densitometric quantification of blots in (F). (H) Representative immunofluorescence staining of TGF-β1 and p-Smad2/3 in BMDMs (n=3 for each group). Scale bar, 50 μm. (I) Quantification of the positive staining area (%) in (H). Data are presented as mean ± SD. *P<0.05, **P<0.01, ***P<0.001, ****P < 0.0001.

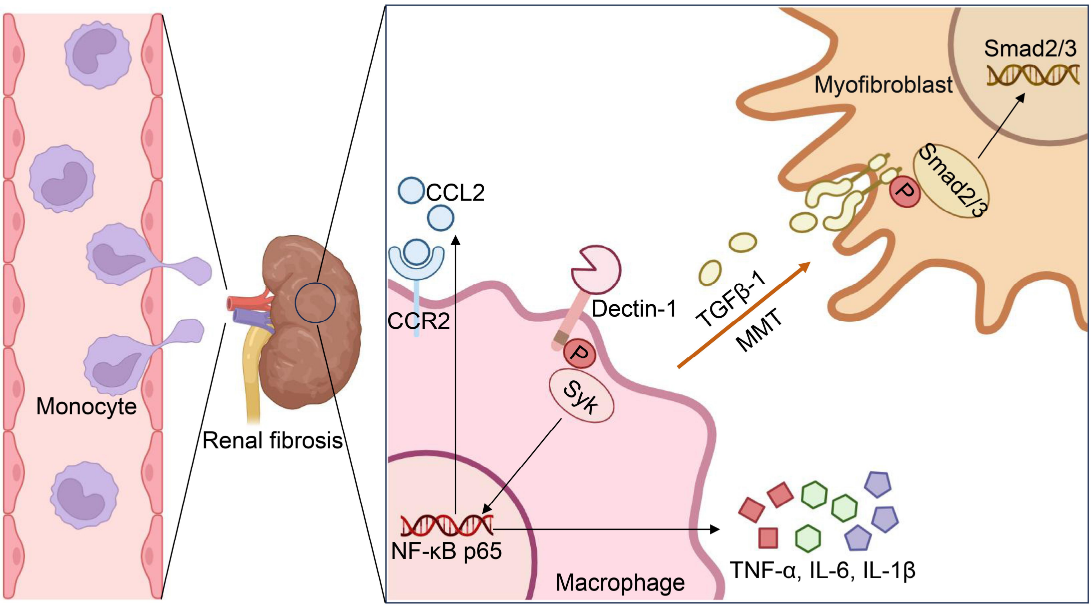

Supplementary Figure S11. Dectin-1 promotes renal fibrosis by enhancing macrophage infiltration via Syk/NF-κB/CCL2-CCR2 axis and facilitating macrophage-to-myofibroblast transition (MMT) through TGF-β/Smad activation.

Supplementary Table S1. Clinical characteristics of IgAN patients.

	T0 (*n* = 42)	T1 (*n* = 42)	*P*
Age (years)	37.38 ± 12.43	41.52 ± 14.43	0.1624
Sex (male)	21 (50%)	28 (66.67%)	0.1213
Serum creatinine (μmol/L)	88.76 ± 24.36	138 ± 43.02	< 0.0001
Blood urea nitrogen (mg/dL)	5.289 ± 1.272	7.595 ± 3.029	< 0.0001
Serum albumin (g/L)	44.15 ± 6.892	38 ± 4.124	< 0.0001
Urinary protein (g/d)	0.8635 ± 0.7368	1.222 ± 0.8916	0.0571
Urinary albumin creatinine ratio	0.6033 ± 0.5271	1.494 ± 1.416	0.0004
eGFR (ml/min/1.73m^2)	87.61 ± 22	54.87 ± 20.53	< 0.0001
Diabetes mellitus (*n*, %)	1 (2.48%)	0	> 0.9999
Hypertension (*n*, %)	9 (21.43%)	22 (52.38%)	0.0033

Supplementary Table S2. Primer sequences used for RT-qPCR.

Gene	Species	Forward Primers	Reverse Primers
Gapdh	Mouse	AGGTCGGTGTGAACGGATTTG	TGTAGACCATGTAGTTGAGGTCA
Clec7a	Mouse	GGGTGCCCTAGGAGGTTTTT	AACCATGGCCCTTCACTCTG
Col1a1	Mouse	GCTCCTCTTAGGGGCCACT	ATTGGGGACCCTTAGGCCAT
Vim	Mouse	CAGAGAGAGGAAGCCGAAAG	ATGCTGTTCCTGAATCTGGG
Fn1	Mouse	ATGTGGACCCCTCCTGATAGT	GCCCAGTGATTTCAGCAAAGG
Acta2	Mouse	CCCAGACATCAGGGAGTAATGG	TCTATCGGATACTTCAGCGTCA
Tgfb1	Mouse	GAGCCCGAAGCGGACTACTA	TGGTTTTCTCATAGATGGCGTT
Ccr2	Mouse	ATCCACGGCATACTATCAACATC	TCGTAGTCATACGGTGTGGTG
Tnf	Mouse	ATGGCCTCCCTCTCATCAGT	TTTGCTACGACGTGGGCTAC
Ccr1	Mouse	AGTAAGCAACTGGACCTGGC	CTTCCAGAACCGTTCACCCA
Ccr5	Mouse	GTTGTTTTGGAGAACGCCCC	CAACACTGCTCCGAAACTGC
Ccr7	Mouse	CATGGACCCAGGTGTGCTTC	TCAGTATCACCAGCCCGTTG
Ccr8	Mouse	TGTTTGGGACTGCGATGTGT	TGATGGCATAGACAGCGTGG
Cxcr4	Mouse	ATGGAACCGATCAGTGTGAG	TCACCAATCCATTGCCGACT
Cxcr6	Mouse	CAGGCACCTATGAGTGGGTC	ATCTTCCACTTAGCCTGCCG
Cx3cr1	Mouse	TCGTCTTCACGTTCGGTCTG	CTCAAGGCCAGGTTCAGGAG
Ccl19	Mouse	ACTTGCACTTGGCTCCTGAAC	AGGCTTTCACGATGTTCCCA
Ccl21	Mouse	ACAGCTGGTGGTAACGAGGAAA	ACTTAAGGCAGCAGTCCTGT
Ccl2	Mouse	CAGGTCCCTGTCATGCTTCT	GTGGGGCGTTAACTGCATCT
Il6	Mouse	CCCCAATTTCCAATGCTCTCC	CGCACTAGGTTTGCCGAGTA
Il1b	Mouse	CAGGCAGGCAGTATCACTCA	AGCTCATATGGGTCCGACAG
Nos2	Mouse	GCCCAGCCAGCCCAAC	GCACATCAAAGCGGCCATAG
Mrc1	Mouse	GTCAGAACAGACTGCGTGGA	AGGGATCGCCTGTTTTCCAG
Arg1	Mouse	AGCACTGAGGAAAGCTGGTC	TACGTCTCGCAAGCCAATGT
GAPDH	Human	GGCATCCTGGGCTACACTGA	GGAGTGGGTGTCGCTGTTG
NGAL	Human	AGTGCACAGGTGCCGC	TTTAGCAGACAAGGTGGGGC
